# An Antibody‐Drug Conjugate for Multiple Myeloma Prepared by Multi‐Arm Linkers

**DOI:** 10.1002/advs.202307852

**Published:** 2024-03-13

**Authors:** Yueh‐Hsiang Yu, Wei‐Ting Tian, Cédric Grauffel, Wei‐Chen Lin, Ming‐Yu Hsieh, Pei‐Wen Wu, Hui‐Ju Lee, Chi‐Jiun Peng, Pei‐Hsuan Lin, Hsing‐Mao Chu, Carmay Lim, Tse Wen Chang

**Affiliations:** ^1^ Immunwork, Inc. Academia Rd., Sec. 1, Nangang Taipei 115 Taiwan; ^2^ Institute of Biomedical Sciences Academia Sinica Academia Rd. Taipei 115 Taiwan

**Keywords:** anti‐CD38, lenalidomide, scFv, site‐specific conjugation, Zn^2+^‐binding motif

## Abstract

First‐line treatment of multiple myeloma, a prevalent blood cancer lacking a cure, using anti‐CD38 daratumumab antibody and lenalidomide is often inadequate due to relapse and severe side effects. To enhance drug safety and efficacy, an antibody‐drug conjugate, TE‐1146, comprising six lenalidomide drug molecules site‐specifically conjugated to a reconfigured daratumumab to deliver cytotoxic lenalidomide to tumor cells is developed. TE‐1146 is prepared using the HighDAR platform, which employs i) a maleimide‐containing “multi‐arm linker” to conjugate multiple drug molecules creating a drug bundle, and ii) a designed peptide with a Zn^2+^‐binding cysteine at the C‐termini of a reconfigured daratumumab for site‐specific drug bundle conjugation. It is shown that TE‐1146 remains intact and effectively enters CD38‐expressing tumor cells, releasing lenalidomide, leading to enhanced cell‐killing effects compared to lenalidomide/daratumumab alone or their combination. This reveals the remarkable potency of lenalidomide once internalized by myeloma cells. TE‐1146 precisely delivers lenalidomide to target CD38‐overexpressing tumor cells. In contrast, lenalidomide without daratumumab cannot easily enter cells, whereas daratumumab without lenalidomide relies on Fc‐dependent effector functions to kill tumor cells.

## Graphical abstract

Uncover the transformative potential of TE‐1146 to treat multiple myeloma. TE‐1146, prepared using an innovative HighDAR platform, comprises six lenalidomide drug molecules site‐specifically conjugated to a reconfigured daratumumab. It shows enhanced target cell‐killing activity compared to daratumumab, alone or combined with lenalidomide, as it delivers lenalidomide specifically to target MM cells. Lenalidomide alone cannot easily enter cells.

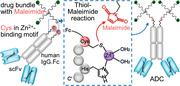

## 1 Introduction

Antibody‐drug conjugates (ADCs) are promising anticancer therapeutics, as evidenced by over 100 ADCs in clinical studies and 12 FDA approvals to date.^[^ ^1^ ^]^ They consist of cytotoxic drugs linked to monoclonal antibodies (mAbs) that specifically target tumor cell‐surface proteins. Once bound to these antigens, the ADC‐antigen complex is internalized into cells, where the linker connecting the mAb and the drug is cleaved, releasing the cytotoxic drug for cell‐killing effects. ADC development faces challenges in producing *homogenous* ADCs with a *high* drug:antibody ratio (DAR) without increasing hydrophobicity/aggregation,^[^ ^2^ ^]^ and preventing premature drug release at off‐target sites, leading to toxicity.^[^ ^3^ ^]^ Here, we introduce HighDAR, a modular platform for producing homogenous, stable, high‐DAR ADCs without requiring enzymes. HighDAR uses a “multi‐arm linker” to bundle multiple drug molecules that are conjugated to a reduced‐size antibody via click chemistry. We demonstrate this platform by preparing a stable DAR‐6 ADC (TE‐1146) targeting CD38 (UniProt: P28907), a protein overexpressed in multiple myeloma (MM) compared to normal cells.^[^ ^4^ ^]^

Early approved ADCs exhibited heterogeneity in DAR, stabilities, pharmacokinetics, antibody‐antigen binding affinities, and efficacy,^[^ ^5^ ^]^ as the cytotoxic drugs are randomly conjugated to native lysines (e.g., inotuzumab ozogamicin/Besponsa/ado‐trastuzumab emtansine/Kadcyla) or cysteines (e.g., sacituzumab govitecan/Trodelvy/fam‐trastuzumab derutecan‐nxki/Enhertu) in the mAb. Although the two approved ADCs, Enhertu and Trodelvy, have a mean DAR of ≈8, it is uncertain if DAR‐8 ADCs for other linker‐payloads formed by breaking interchain S‐S bonds, which contribute to antibody stability, can maintain in vivo stability.^[^ ^5^, ^6^ ^]^ Indeed, DAR‐8 ADCs resulting from reducing 4 interchain disulfide bonds have shown reduced circulation half‐lives or maximal tolerable doses compared to DAR‐4 ADCs.^[^ ^7^ ^]^

To produce homogenous ADCs with identical DAR and drug‐conjugation sites, various site‐specific conjugation methods have been developed using chemical, enzymatic, and/or genetic approaches.^[^ ^1^, ^2^, ^5^, ^8^ ^]^ These methods involve equipping the antibody with unique reactive groups such as 1) solvent‐exposed Cys thiols,^[^ ^9^ ^]^ 2) engineered amino acid (aa) residues or peptides,^[^ ^10^ ^]^ 3) unnatural/non‐canonical aa residues with reactive groups,^[^ ^6^, ^11^ ^]^ or 4) modified glycan^[^ ^12^ ^]^ for site‐specific drug conjugation. However, non‐native Cys may destabilize the antibody, modified or unnatural aa sequences can induce immunogenicity and antibody aggregation, and enzymatic alterations may inadvertently affect native residues in the antibody. Current site‐specific methods typically yield ADCs with DAR ≤ 4.^[^ ^1^, ^5^, ^6^ ^]^ Moreover, the large size of IgG‐based ADCs can hamper effective penetration into solid tumors.^[^ ^13^ ^]^

Here, we introduce our HighDAR platform for preparing homogeneous, stable ADCs with DAR > 4. This platform incorporates 3 novel constructs; viz., 1) a maleimide‐containing “multi‐arm linker” to attach drug molecules and couple the “drug bundle” to an antibody via an SH‐maleimide reaction, 2) a 2‐chain scFv‐Fc antibody (instead of a 4‐chain IgG antibody) to reduce two disulfide bonds and the protein size, enabling better tumor penetration, and 3) A designed 6‐mer peptide with a Zn^2+^‐binding cysteine − the Zn^2+^ protects the Cys from unwanted disulfide bond formation and activates it for reaction with maleimide.^[^ ^5^, ^14^ ^]^

To preserve the antibody's structure and antigen‐binding ability, we attach the Zn^2+^‐binding motif to the C‐terminus of each scFv‐Fc chain using a flexible (Gly)_3_‐linker. By incorporating a specific number of drugs in the “multi‐arm linker”, our platform can generate ADCs with higher DAR values compared to current site‐specific conjugation methods.^[^ ^1^, ^5^ ^]^

We aimed to use our platform to develop an ADC to address the unmet need for effective MM therapy. MM is a neoplastic malignancy characterized by abnormal plasma cell proliferation and excessive antibody production in the bone marrow. It is considered incurable^[^ ^15^ ^]^ and accounts for ≈1.8% of new cancer cases and 2.1% of cancer‐related deaths in the United States.^[^ ^16^ ^]^ Frequently used MM treatments, daratumumab (a human IgG1κ mAb targeting CD38, CAS number: 945721‐28‐8),^[^ ^17^ ^]^ and lenalidomide (an immune regulator), either alone or in combination,^[^ ^18^ ^]^ show limitations. Around 50% of MM patients only achieve partial responses or fail to respond to the combination therapy, and patients often experience severe side effects such as neutropenia, anemia, thrombocytopenia, diarrhea, fatigue, and upper respiration tract infections.^[^ ^19^ ^]^ Recent MM treatments, including two CAR‐T therapies targeting B‐cell maturation antigen (BCMA) (idecabtagene vicleucel/Abecma, ciltacabtagene autoleucel/Carvykti) and three bispecific T‐cell engagers targeting CD3 and BCMA (teclistamab/Tecvayli, elranatamab/Elrexfio) or G protein‐coupled receptor family C, group 5 member D (talquetamab/Talvey), are associated with severe side effects. These include cytokine release syndrome, hematologic and neurologic toxicity, and the risk of infection.^[^ ^20^ ^]^ An antibody fusion protein drug comprising two interferon α2β molecules fused to an anti‐CD38 mAb (modakafusp alfa/TAK‐573) in phase 1/2 studies remains to be fully evaluated for safety and tolerability in patients with MM and relapsed/refractory MM (https://clinicaltrials.gov/). The DAR‐4 ADC (Belantamab mafodotin/Blenrep), approved by the U.S. FDA in 2020, was withdrawn in 2022. Several of the ADCs developed for treating MM have been discontinued, whereas the remaining have DAR ≤4. Thus, we have developed a DAR‐6 ADC (TE‐1146) comprising a reconfigured daratumumab conjugated to 2 drug bundles, each containing 3 lenalidomide molecules, via the C‐terminal Zn^2+^‐binding Cys.

We show that TE‐1146 remains intact in human plasma and effectively enters MM cells, releasing lenalidomide, resulting in enhanced cell‐killing effects compared to lenalidomide/daratumumab alone or their combination. In NOD‐SCID mice with transplanted H929 cells, a single dose of TE‐1146 significantly slowed tumor growth and eventually eliminated the transplanted tumor over 28−42 days. In contrast, daily injections of daratumumab and lenalidomide failed to eliminate the tumor. Notably, the amount of lenalidomide used in the combination treatment over 28 days exceeded that in TE‐1146 by a factor of 10 700. Our findings underscore the remarkable potency of lenalidomide once internalized by myeloma cells. TE‐1146 exhibits enhanced efficacy in killing MM cells in both cell culture and in vivo animal models due to its ability to deliver lenalidomide directly to target cells.

## 2 Results

Below, we first describe the rationale design, production, and characterization of TE‐1146, a *homogenous* DAR‐6 ADC, and then the results of in vitro studies followed by in vivo studies.

### 2.1 Zn^2+^ Binds and Activates the C‐Terminal Cys to React with Maleimide

**Figure 1:**
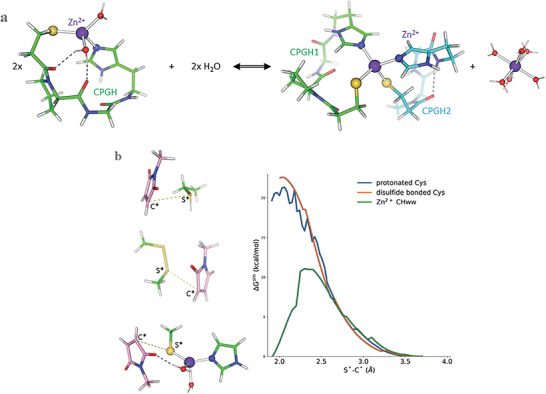
(Top) Formation of a single [Zn^+^−CCHH]^0^ site from two [Zn−CHww]^+^ motifs in solution. The computed formation free energy in solution, *Δ*G^sln^, is 17.4 kcal mol^−1^, whereas the corresponding entropy multiplied by temperature, *Δ*S^sln^×T, is −18.3 kcal mol^−1^. (Bottom) Solution free energy *Δ*G^sln^ profiles for maleimide to form an S−C bond with a) protonated Cys (blue curve), b) disulfide‐bonded Cys (orange curve), and (c) Zn^2+^−CHww motif (green curve).

To conjugate the maleimide‐containing drug bundle to an α‐CD38 mAb, we designed a Zn^2+^‐binding A**C**PG**H**A peptide. The binding of Zn^2+^ induces deprotonation of the cysteine to a reactive thiolate,^[^ ^21^ ^]^ protecting the cysteine from unwanted disulfide bond formation − a critical distinction from cysteines incorporated in an antibody that typically remain protonated at physiological pH and may form disulfide bonds with substances in the surrounding medium. To elucidate if the A**C**PG**H** peptides bind Zn^2+^ to form (i) two “wet” Zn^2+^−CHww sites (w = water) or (ii) one “dry” Zn^2+^−CCHH site, we computed the free energy for forming a single Zn^2+^−CCHH site from two Zn^2+^−CHww sites in water (**Figure** **1**a). The calculations show that the formation of a single *rigid* [Zn^+^−CCHH]^0^ site from two *flexible* [Zn−CHww]^+^ sites results in a significant entropy loss, making it thermodynamically unfavorable. Next, we confirmed that Zn^2+^‐bound Cys^−^ reacts more readily with maleimide compared to free or disulfide‐bonded Cys by computing solution free energy profiles for i) Zn^2+^‐bound Cys^−^, ii) protonated Cys, and iii) disulfide‐bonded Cys to form an S−C bond with maleimide. The results in Figure 1b reveal that the reaction barrier for deprotonated Cys^−^ is roughly half that for protonated or disulfide‐bonded Cys, confirming that Zn^2+^ activates the C‐terminal Cys for maleimide attack.

### 2.2 Creating Lenalidomide Drug Bundles using a Multi‐Arm Linker

**Figure 2:**
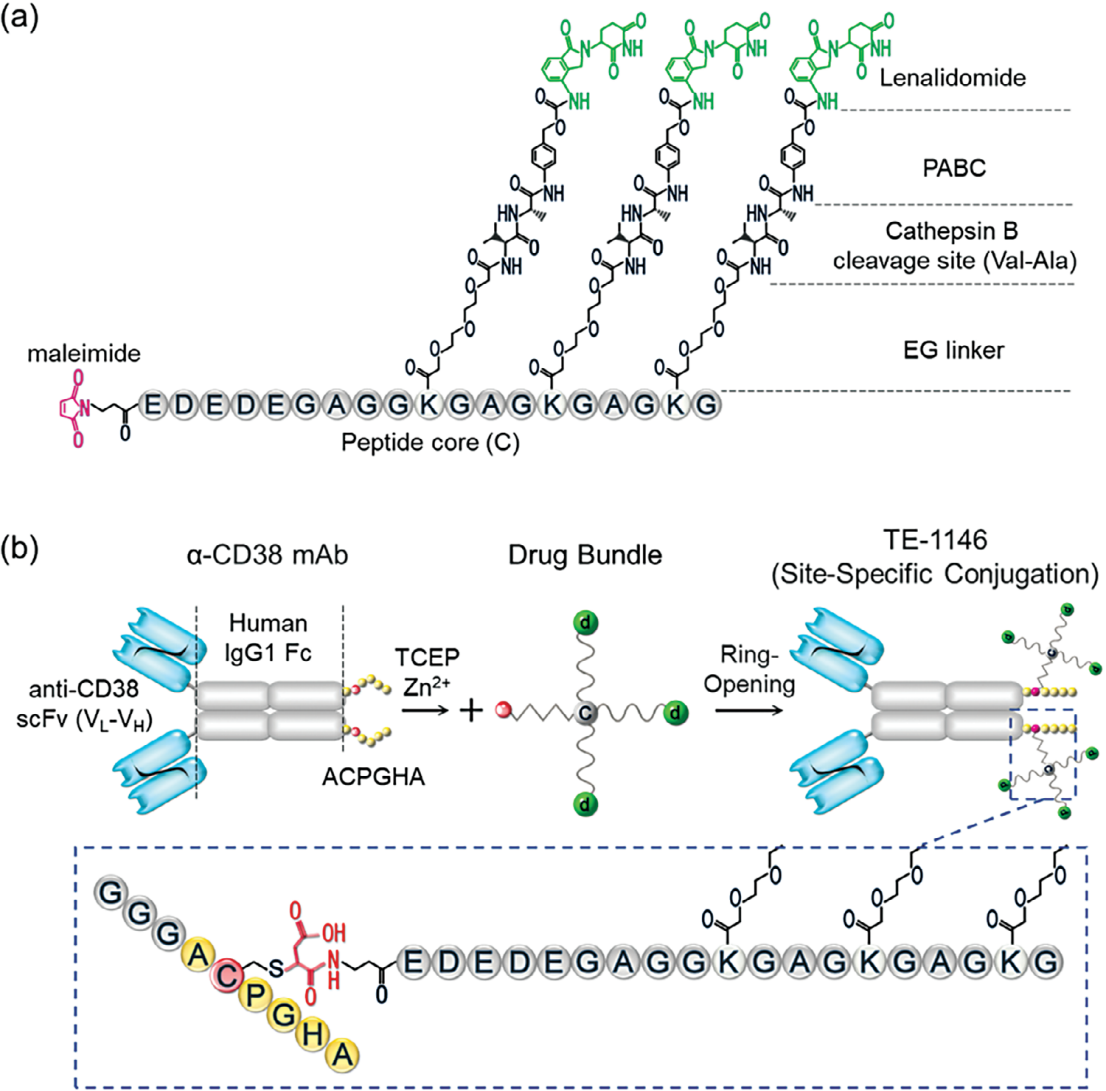
Preparing and conjugating drug bundles to α‐CD38 mAb. a) Illustration of a lenalidomide drug bundle produced by a “multi‐arm linker”. b) Fusion of scFv α‐CD38 (blue) and Zn^2+^‐binding motifs (yellow) to IgG1.Fc (grey) yields an α‐CD38 fusion protein. The drug bundle consists of a peptide core c), a maleimide group (red), and three lenalidomide drug molecules d) linked to cleavable linking arms (wavy lines) recognized by cathepsin B. TE‐1146 is formed by S^−^‐maleimide reaction and irreversible hydrolysis of the succinimidyl moiety, resulting in ring opening.

The multi‐arm linker used to construct the drug bundle consists of three components (**Figure** **2**a):

1) A *coupling arm* using a maleimido‐ethyl‐CO_2_H group to couple the drug bundle to the engineered Zn^2+^‐binding Cys of the α‐CD38 antibody. 2) *Linking arms*, each comprising an ethylene glycol (EG) linker, a Val‐Ala dipeptide recognized by cathepsin B, and para‐aminobenzylcarbamate (PABC) to link lenalidomide. 3) A *peptide core*, NH_2‐_(ED)_2_EGAGG(**K**GAG)_2_ **K**G‐COOH, linked to the coupling arm at the N‐terminus with acidic residues to enhance solubility and small residues between the Lys to provide adequate space for the linking arms extending from the Lys ε‐amino groups.

The synthesized lenalidomide drug bundle was purified to ≈95% purity, as determined by liquid chromatography (LC)‐mass spectrometry (MS) analysis. It displayed a molecular ion at 1379.2, corresponding to [M+3H]^3+^ (Figure S1, Supporting Information), indicating a molecular weight (MW) of ≈4135 Da for the drug bundle, consistent with the calculated MW.

### 2.3 Linking Drug Bundles to α‐CD38 mAb Yielding DAR‐6 TE‐1146

To produce TE‐1146, genes encoding a reconfigured anti‐CD38 scFv‐Fc with a Zn^2+^‐binding ACPGHA motif were synthesized and expressed in CHO cells. This yielded an α‐CD38 fusion protein comprising two [(scFv α‐CD38)‐CH2‐CH3‐(Gly)_3_‐ACPGHA] chains. Following purification, the antibody was treated with a low concentration of tris (2‐carboxyethyl) phosphine (TCEP) to free the Zn^2+^‐binding Cys from unwanted disulfide bonds and mixed with Zn^2+^ to generate reactive Zn^2+^‐bound thiolates. Upon adding the maleimide‐containing drug bundles, the Zn^2+^‐bound thiolates reacted with the maleimide, forming thiosuccinimide conjugates with 3.5% high MW aggregation (Figure 2b). To prevent premature drug bundle release stemming from exchange reactions with plasma/blood thiols (e.g., human serum albumin or glutathione),^[^ ^22^ ^]^ the thiosuccinimide was hydrolyzed under alkaline conditions to stabilize the ADC through ring opening (Figure 2b, inset). The percentage of high MW aggregation increased to 10.4% after hydrolysis and 18.3% after buffer exchange (Figure S2, Supporting Information). Fractionation using hydrophobic interaction column (HIC) and size exclusion chromatography (SEC) removed nearly all high MW aggregation, resulting in TE‐1146 purity exceeding 95%, as quantified by SDS‐PAGE, HIC‐HPLC, and SEC‐HPLC (Figure S3a–c, Supporting Information).

After deconvolution, LC‐MS analysis revealed that half of the purified TE‐1146 (a single chain with one drug bundle) has a MW of 56 979 Da. In MALDI‐TOF analysis, the observed mass‐to‐charge (m/z) values of the singly (Z = 1) and doubly (Z = 2) charged TE‐1146 were 116 820 and 58 501 Da, respectively, whereas the drug‐free α‐CD38 exhibited m/z values of 108 534 (Z = 1) and 54 311 Da (Z = 2) (Figure S4a,b, Supporting Information). Since the MW of the drug bundle is 4135 Da, the MW increase of 8286 Da in TE‐1146 compared to unconjugated α‐CD38 for the Z = 1 species indicates the presence of two drug bundles. Hence, TE‐1146 has a DAR of 6.

### 2.4 Drug Bundles Conjugate to the C‐Terminal Cys and did not Affect Antigen Binding

**Figure 3:**
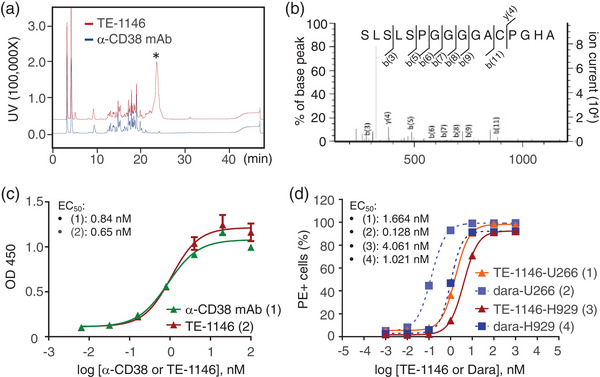
Drug conjugation site and antigen‐binding of TE‐1146. a) TE‐1146 (red) and α‐CD38 (blue) underwent trypsin digestion, and the resulting peptides were analyzed by HPLC. An additional peak in the trypsin‐digested TE‐1146 profile is highlighted with an asterisk (*). b) The aa sequence of the peptide fragment containing the engineered Cys is shown above the spectrum. The y‐ion, y(4), and the b‐ions, b(3,5,6,7,8,9,11), are indicated above and below the sequence, respectively. c) The binding affinities of α‐CD38 and TE‐1146 to human CD38 were assessed via a cell‐based enzyme‐linked immunosorbent assay (ELISA) using a human CD38‐overexpressing 293T cell line. d) The binding abilities of TE‐1146 (solid curves) and daratumumab (dashed curves) to cell surface‐expressed CD38 were characterized by flow cytometry using U266‐CD38^+^ and H929 MM cell lines. The EC_50_, the concentration of each protein resulting in 50% of the maximal response of binding to target cells, is shown.

To confirm site‐specific drug bundle conjugation, TE‐1146 was reduced and treated with iodoacetamide, which adds a methylcarboxyamido group (CH_2_CONH_2_) to unconjugated Cys(S). LC‐Electrospray Ionization (ESI)‐MS analysis of trypsin‐digested TE‐1146 peptide segments revealed six Cys‐containing peptide segments, among which only the C‐terminal SLSLSPGGGGA **C** PGHA segment exhibited drug bundle conjugation: The observed m/z of 5571.3 matching the theoretical m/z (Figure S5a–g, Supporting Information). To confirm the drug conjugation site, unconjugated α‐CD38, and drug‐conjugated TE‐1146 underwent trypsin digestion and subsequent HPLC analysis. A comparison of their HPLC profiles revealed an additional peak in TE‐1146 (**Figure** **3**a). MS/MS analysis of this peak showed it to be the ^472^SLSLSPGGGGA **C** PGHA^487^ segment, where the Cys was conjugated to a lenalidomide bundle. In the collision‐induced dissociation profile of this fraction (Figure 3b), the matched y‐ion represented the peptide with an identical MW of the indicated sequence from the marked site to the C‐terminal end, whereas the b‐ion represented the peptide with an exact MW of the indicated sequence from the N‐terminal end to the marked site. The mass of the y‐ or b‐ion matched the corresponding theoretical mass of the C‐terminal peptide fragment.

To confirm that the attachment of two drug bundles to the antibody's C‐termini did not alter its CD38‐binding ability, we assessed the binding activities of α‐CD38 and TE‐1146 to human CD38 by ELISA. Conjugating six drug molecules to α‐CD38 did not compromise the antibody's targeting functionality: Both α‐CD38 and drug‐conjugated TE‐1146 showed similar binding affinities to human CD38, with EC_50_ values of 0.84 and 0.65 nM, respectively (Figure 3c).

We also assessed the binding affinity of TE‐1146 to live human CD38^+^ MM cells via flow cytometric analysis. We chose commonly used human MM cell lines for MM investigation, namely, H929 and MM.1S, which express CD38 and are lysed by daratumumab‐mediated immunological mechanisms.^[^ ^23^ ^]^ Note that MM.1S cells are known to express lower baseline CD38 levels than H929 MM cells.^[^ ^24^ ^]^ We also employed the human MM cell line, U266, which has two subtypes: U266‐CD38^+^ cells expressing CD38 and U266‐CD38^−^ cells lacking CD38 expression. TE‐1146, like its “parent” daratumumab, showed specific and dose‐dependent binding to U266‐CD38^+^ and H929 cells (Figure 3d) as well as MM.1S cells, but not to U266‐CD38^−^ MM cells (Figure S6a–c, Supporting Information). However, TE‐1146 displayed higher EC_50_ values for binding to U266‐CD38^+^ (1.66 nM), MM.1S (2.64 nM), and H929 (4.06 nM) compared to daratumumab (0.13, 0.58, and 1.02 nM, respectively), indicating weaker affinity for human CD38 than daratumumab. Additionally, cell‐free ELISA experiments revealed that TE‐1146 exhibited weaker binding to recombinant human CD38 proteins compared to daratumumab. This suggests that the weaker binding of TE‐1146 to CD38^+^ MM cells compared to daratumumab is not due to anion‐anion repulsion between acidic residues on the multi‐arm linker and the cell surface. Instead, it may be linked to conformational differences resulting from the replacement of daratumumab's anti‐CD38 IgG with a scFv‐IgG.Fc in TE‐1146.

### 2.5 TE‐1146 is Stable in Human Plasma

**Figure 4:**
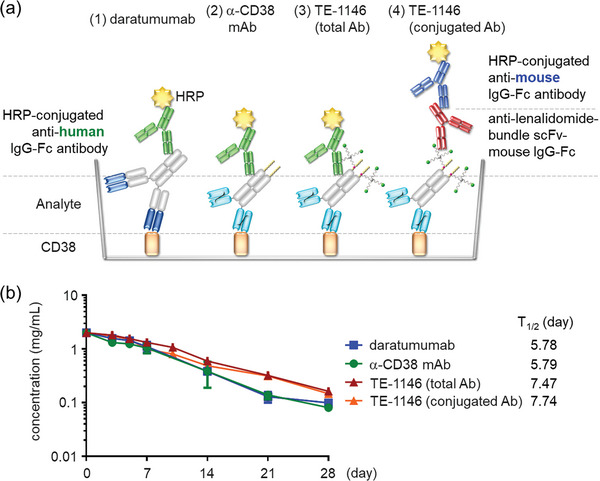
Plasma stability of TE‐1146. a) HRP (yellow) conjugated to anti‐human IgG‐Fc (green) was used to detect 1) daratumumab, 2) α‐CD38 mAb, and 3) total TE‐1146 (total Ab), regardless of whether the drug bundles remain conjugated to TE‐1146. To determine if the 2 lenalidomide bundles remain conjugated to TE‐1146 (conjugated Ab), anti‐lenalidomide‐bundle scFv‐mouse IgG‐Fc (red) was used to detect the conjugated lenalidomide bundle, and it, in turn, was detected by a secondary antibody, HRP‐anti‐mouse IgG‐Fc (blue). b) The stability of daratumumab, α‐CD38 mAb, or TE‐1146 incubated in human plasma for 28 days determined by ELISA. Data are shown as mean ± SD.

We assessed the stability of TE‐1146 compared to that of daratumumab or unconjugated α‐CD38. All three molecules were dissolved in 90% human plasma, incubated at 37 °C for 28 days, and analyzed by ELISA. Daratumumab, α‐CD38 mAb, and TE‐1146 exhibited similar half‐lives (T_1/2_) of ≈6−7 days in human plasma over the 28 days. However, the secondary antibody, HRP‐conjugated anti‐human IgG‐Fc antibody, used in the ELISA (**Figure** **4**a) detected total TE‐1146 without distinguishing between TE‐1146 conjugated to two drug bundles and those with ≤1 drug bundle. To determine if the lenalidomide bundles remained attached to TE‐1146, an anti‐lenalidomide‐bundle scFv‐mouse IgG‐Fc antibody was constructed and detected by a secondary antibody, HRP‐anti‐mouse IgG‐Fc antibody. The results in Figure 4b showed that the 2 lenalidomide bundles remained stably conjugated to TE‐1146 in human plasma (denoted as conjugated Ab): The T_1/2_ of the lenalidomide bundle‐conjugated Ab (7.7 days) was similar to that of total TE‐1146 (7.5 days). Furthermore, despite the reduced scFv in TE‐1146, its T_1/2_ is comparable to daratumumab's T_1/2_ of ≈6 days.

### 2.6 TE‐1146 Enables Intracellular Lenalidomide Release

**Figure 5:**
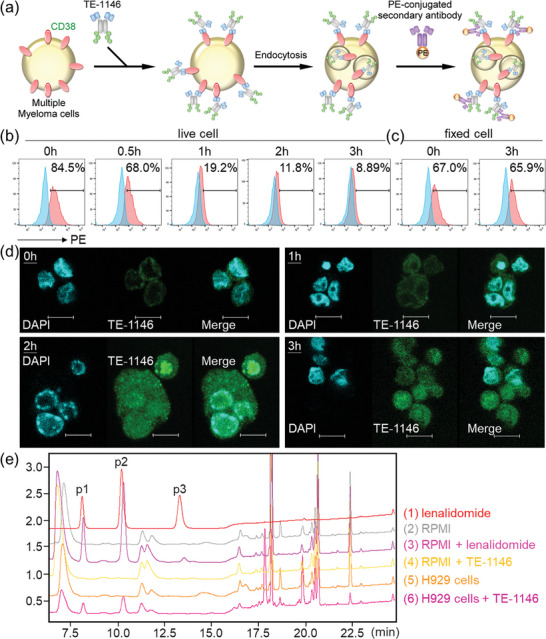
Internalization of TE‐1146 and subsequent lenalidomide release from drug bundles. a) The procedure to determine the ratio of TE‐1146 internalization into MM cells. Flow cytometry analysis of the degree of TE‐1146 internalization at indicated time points in b) live H929 cells and c) H929 cells fixed with 100% ethanol to abolish the internalization process. d) Confocal microscopy analysis of the internalization of TE‐1146 conjugated with VivoTag 645 at different incubated times (0, 1, 2, and 3 h). Upon binding to DNA AT regions, DAPI (4′,6‐diamidino‐2‐phenylindole) emits blue fluorescence and is used to detect cell nuclei. e) HPLC detection of lenalidomide release from TE‐1146 after incubating with MM cells. 1) lenalidomide in phosphate‐buffered saline (PBS); 2) RPMI medium; 3) lenalidomide in RPMI medium; 4) TE‐1146 in RPMI medium; 5) 5‐day cell culture supernatant of H929 cells; 6) 5‐day cell culture supernatant collected from 1 × 10^6^ H929 cells incubated with TE‐1146.

To investigate lenalidomide release from TE‐1146 upon cellular entry, we conducted two internalization assays to distinguish between internalization and dissociation of TE‐1146. Parallel experiments were performed with i) H929 cells fixed with 100% ethanol to prevent internalization and ii) live, unfixed H929 cells. First, H929 cells were incubated with a saturating level of TE‐1146 on ice for 30 min to halt the internalization of CD38‐bound TE‐1146 on the cell surface. After washing, the TE‐1146‐bound H929 cells were incubated for a further 0.5, 1, 2, or 3 h at 37 °C, and the TE‐1146 molecules remaining on the cell surface were labeled with phycoerythrin (PE)‐conjugated anti‐human IgG‐Fc antibody and detected using flow cytometry (**Figure** **5**a). In live H929 cells, the signal intensity of TE‐1146 decreased by ≈15% after 30 min and by an additional 60% after 3 h (Figure 5b), indicating that fewer TE‐1146 molecules remained bound to the cell surface. Importantly, this signal intensity decrease was not due to TE‐1146 dissociation during incubation, because fixed cells displayed no loss of TE‐1146 signal intensity after 3 h of TE‐1146 incubation (Figure 5c).

We also assessed TE‐1146 internalization by H929 cells using confocal microscopy. TE‐1146, labeled with the fluorescent dye, VivoTag 645, exhibited comparable binding activity to that of TE‐1146 (Figure S7a, Supporting Information). Confocal images revealed substantial accumulation of TE‐1146 inside the cells after 2−3 h of incubation with TE‐1146, indicating that TE‐1146, upon binding to CD38 on the cell surface, undergoes cellular internalization within 2 h (Figure 5d).

Next, we confirmed lenalidomide release from TE‐1146 upon cathepsin B cleavage in the lysosome after TE‐1146 internalization by H929 cells using HPLC. Figure 5e provides evidence for lenalidomide release from TE‐1146 after incubating with MM cells: Lenalidomide in PBS (red curve) or RPMI medium (pink curve) is characterized by three peaks (p3, p2, and p1). LC–MS analysis showed that the p3 peak corresponds to lenalidomide, whereas the p1 and p2 peaks correspond to the two hydrolyzed states of lenalidomide (Figure S8, Supporting Information ). Hydrolyzed lenalidomide (p1 and p2), but not lenalidomide (no p3), was found in culture supernatants of H929 cells incubated with TE‐1146 for 5 days (bottommost magenta curve), confirming the release of lenalidomide from the drug bundles within MM cells. No lenalidomide peaks were detected in RPMI medium (grey curve), TE‐1146 in RPMI (yellow curve), or 5‐day cell culture supernatant of H929 cells (orange curve).

### 2.7 TE1146 is more Cytotoxic to MM cells than the Daratumumab/Lenalidomide Combo

**Figure 6:**
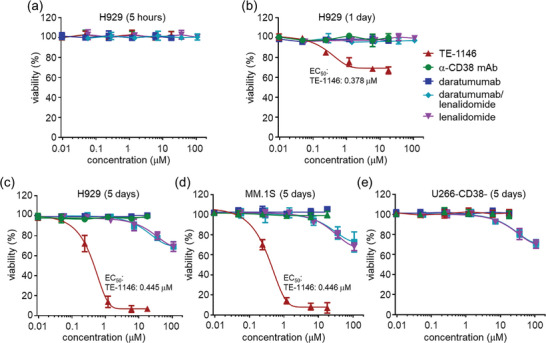
In vitro cytotoxicity of TE‐1146 to MM cells. The percentage of cell viability of H929 cells after 5 h a), 1 day b), and 5 days c) incubation as well as that of MM.1S d), and U266‐CD38^−^ e) cells as a function of increasing concentrations of 5 drugs; viz, TE‐1146 (red), α‐CD38 mAb (green), daratumumab (blue), daratumumab/lenalidomide combination (light blue), and lenalidomide (purple). The EC_50_ values of TE‐1146 are shown. Data are shown as mean ± SD.

We compared the in vitro tumor cell‐killing effects of TE‐1146 with unconjugated α‐CD38 mAb and 3 anti‐MM drugs: i) daratumumab, ii) daratumumab combined with lenalidomide at a 1:6 molar ratio, analogous to the α‐CD38 mAb combined with 6 lenalidomide molecules in TE‐1146, and iii) lenalidomide (equal to the amount of lenalidomide used in the daratumumab/lenalidomide combination). After incubating various MM cells with different drug concentrations for varying durations, the percentage of cell viability was measured. TE‐1146 displayed dose‐dependent cytotoxicity with an EC_50_ of ≈0.45 µM for H929 and MM.1S cells but exhibited no killing effect for U266‐CD38^−^ cells (**Figure** **6**a–e). Interestingly, the EC_50_ of TE‐1146 fell within the EC_50_ range (0.03−0.65 µM) of STI‐6129, an anti‐CD38 antibody conjugated to a microtubule inhibitor (duostatin‐5.2) via a non‐polyethylene glycol linker.^[^ ^25^ ^]^ In contrast, α‐CD38 mAb and parental daratumumab did not induce cell death, even at doses > 20 µM, as their cell‐killing effects relied on other immune cells such as natural killer cells.^[^ ^17^ ^]^ Lenalidomide alone or in combination with daratumumab showed only weak cytotoxicity at concentrations > 10 µM, suggesting limited drug uptake by cells. However, once inside the cells, lenalidomide showed cytotoxic even for U266‐CD38^−^ cells. Despite releasing hydrolyzed lenalidomide rather than lenalidomide itself, TE‐1146 was >100 times more potent than lenalidomide alone or in combination with daratumumab.

### 2.8 Mechanisms of Action: TE1146 kills MM Cells by Releasing Lenalidomide and Inducing Immune Effects

We hypothesized that TE‐1146 is more effective in killing CD38^+^‐MM cells than the daratumumab/lenalidomide combination (Figure 6a–e), because it binds to CD38 and releases the lenalidomide inside the MM cells, whereas the daratumumab/lenalidomide combination lacks this intracellular action. To test this hypothesis, we compared the abilities of the Fc domain of TE1146 and daratumumab in mediating antibody‐dependent cell‐mediated cytotoxicity (ADCC) and complement‐dependent cytotoxicity (CDC) against various MM cells by incubating them with serial dilutions of TE‐1146 or daratumumab in the presence of i) peripheral blood mononuclear cells (PBMCs) containing human immune cells to assess ADCC or ii) human plasma containing complement proteins to assess CDC. Cytolysis was determined by measuring lactate dehydrogenase (LDH) release.

**Figure 7:**
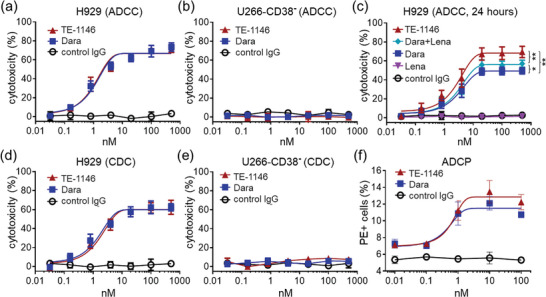
Fc‐mediated immune effects of TE‐1146 to MM cells. a) With short time (5 h) incubation with H929 cells, TE‐1146, and daratumumab (Dara) show comparable ADCC activities. Control IgG was used as the negative control. b) Without CD38 expression, no viability loss of U266‐CD38^−^ cell or ADCC activity was observed after TE‐1146 or Dara incubation for 5 h. c) With long‐term incubation (24 h), TE‐1146 shows a better ADCC effect than Dara, lenalidomide (Lena), or combo treatments. d) Comparable CDC activities of TE‐1146 and Dara were observed after 5 h of incubation with H929 cells. e) TE‐1146 and Dara cannot trigger CDC activity without the expression of CD38 on U266‐CD38^−^ cells. f) Comparable ADCP activities of TE‐1146 and Dara were observed after 5 h of incubation with H929‐eGFP and THP‐1 cells. *p < 0.05; **p < 0.01. Data are shown as mean ± SD.

After 5 h of incubation, TE‐1146 and daratumumab induced similar dose‐dependent ADCC for H929, MM.1S, U266‐CD38^+^, and Daudi cells when PBMCs were present (**Figure** **7**a; Figure S9a–c, Supporting Information). However, no ADCC was observed for U266‐CD38^−^ cells (Figure 7b) or in the absence of PBMCs (Figure 6a). When the incubation extended to one day or more, TE‐1146 exhibited better killing effects than the daratumumab/lenalidomide combination, which, in turn, was more effective than daratumumab alone (Figure 6b). This can be attributed to the initiation of TE‐1146 internalization, resulting in lenalidomide release and MM cell cytotoxicity and lenalidomide's immunomodulating effect, while the ADCC effect was attenuated due to declining PBMCs (Figure 7c; Figure S9d, Supporting Information). Likewise, after 5 h of incubation, TE‐1146 and daratumumab induced similar dose‐dependent CDC for H929, U266‐CD38^+^, and Daudi cells (Figure 7d; Figure S9e,f, Supporting Information), but no CDC was observed for U266‐CD38^− ^cells (Figure 7e).

We also assessed antibody‐dependent cellular phagocytosis (ADCP) by labeling H929 cells and phagocytotic cells (THP‐1, a monocyte‐derived cell line) with eGFP and allophycocyanin (APC) fluorescent dyes, respectively. When THP‐1 cells interacted with the Fc region of CD38‐bound TE‐1146 or daratumumab via Fc receptors, they engulfed H929, resulting in both eGFP and APC signals (Figure S10a–c, Supporting Information). Flow cytometry quantified the proportion of cells exhibiting both signals in the cell culture subjected to different drugs. Both TE‐1146 and daratumumab induced comparable ADCP activities (Figure 7f).

### 2.9 TE‐1146 is Stable and Targets Tumors In Vivo

**Figure 8:**
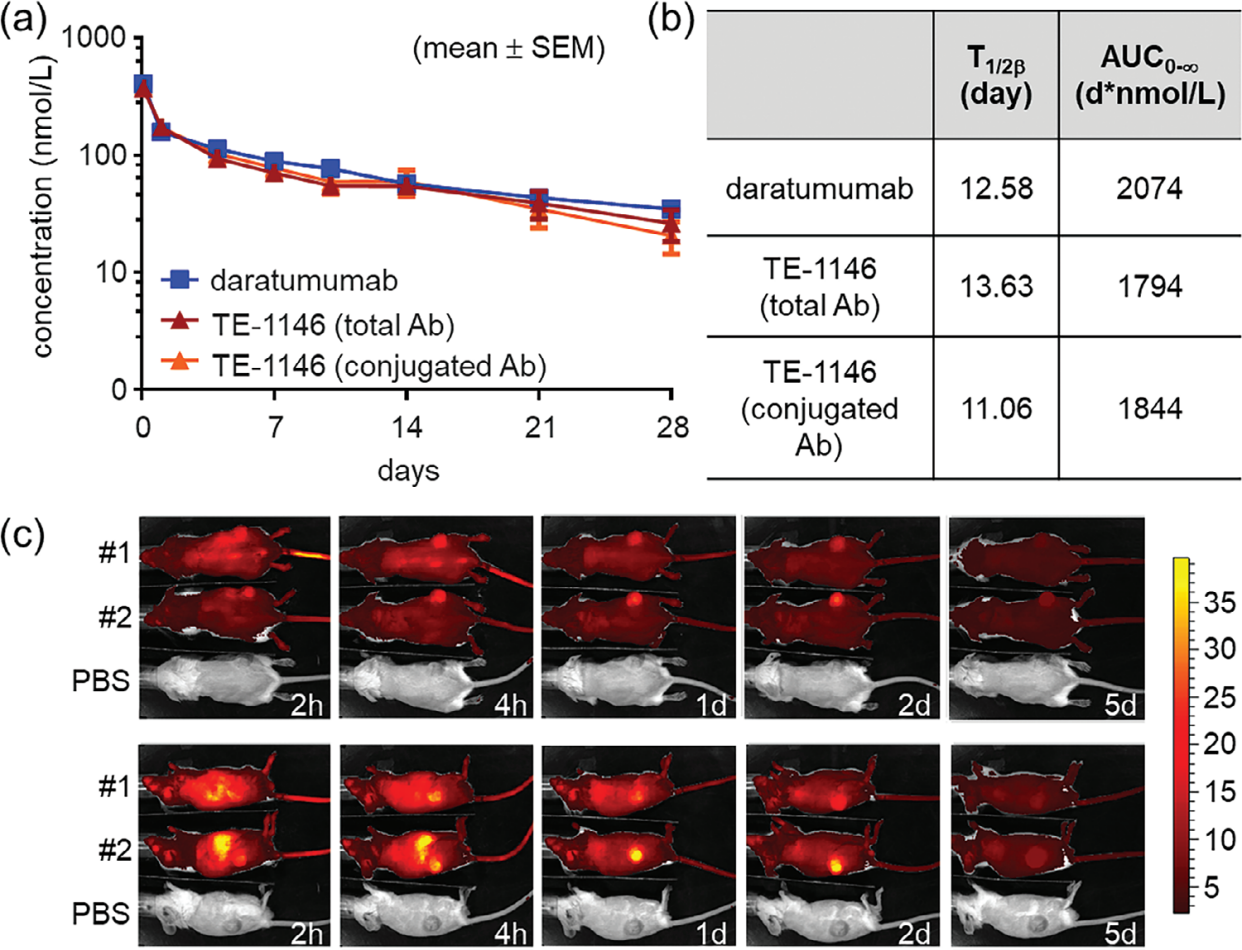
Pharmacokinetics and tumor targeting of TE‐1146. a) Pharmacokinetics of daratumumab and TE‐1146 in C57BL/6J mice in a 28‐day single‐dose study using ELISA to determine mean plasma concentrations of TE‐1146 and daratumumab. b) T_1/2β_ of daratumumab and TE‐1146 at the elimination phase in mouse circulation. c) Back‐side (top) and right‐side (bottom) IVIS imaging of DyLight^TM^ 680‐labeled TE‐1146 in H929 tumor‐bearing mice after 2 h, 4 h, 1 day, 2 days, and 5 days intravenous injection. Data are shown as mean ± SEM.

The pharmacokinetics of TE‐1146 and daratumumab were evaluated using a single intravenous dose in C57BL/6J mice. A semi‐logarithmic plot of the mean plasma concentrations of TE‐1146 (total Ab), TE‐1146 (conjugated Ab), and daratumumab over 28 days revealed similar profiles and comparable elimination phase half‐lives (T_1/2β_) for TE‐1146 (total and conjugated Ab) and daratumumab (**Figure** **8**a,b). Hence, drug bundle conjugation did not impact clearance during the elimination phase, and payload loss due to premature linker cleavage was insignificant. Furthermore, no antibody response to the antibody moiety or the lenalidomide bundle was detected in sera collected for up to 56 days.

To assess the tumor‐targeting efficacy of TE‐1146, we conducted in vivo imaging of DyLight 680‐labeled TE‐1146 in H929 tumor‐bearing mice. The binding affinities of DyLight 680‐labeled TE‐1146 and unmodified TE‐1146 to human CD38 were confirmed to be comparable using ELISA (Figure S7b, Supporting Information). Subsequent IVIS imaging revealed a noticeable concentration of DyLight 680‐labeled TE‐1146 signal at tumor sites as early as 2 h post‐administration, peaking at day 2, and remaining detectable in both back and right‐side IVIS images until day 5 (Figure 8c). In contrast, signals in other body regions had vanished, confirming TE‐1146′s specific targeting and accumulation in MM tumors.

### 2.10 TE‐1146 can Eradicate MM Tumors in Mouse Xenograft Tumor Models

We evaluated TE‐1146′s effectiveness against MM tumors in xenograft mouse models. H929 or MM.1S cells were transplanted in NOD‐SCID mice, and after allowing 14 days for tumors to reach an average volume of 150 ± 20 mm^3^, various treatments were administered: Lenalidomide was administered daily via intraperitoneal injection at its maximum non‐precipitating concentration in the dosing solution, which amounted to 46 µmol kg^−1^. Previous studies on daratumumab's efficacy had employed a single dose of 20 nmol kg^−1^,^[^ ^23^ ^]^ so we adopted this as the initial dose for daratumumab, administered alone or in combination with 46 µmol kg^−1^ day^−1^ of lenalidomide. Additionally, mice received a fixed single dose of TE‐1146 at 20 nmol kg^−1^ or PBS as control.

**Figure 9:**
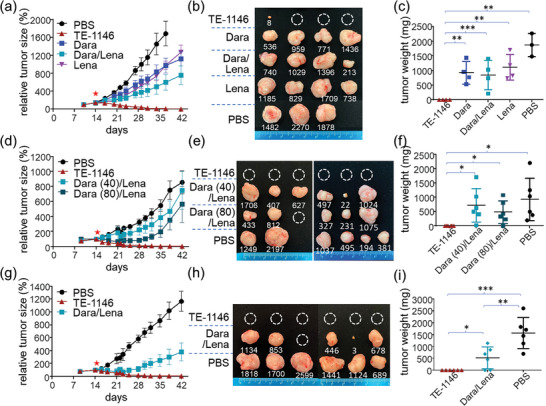
TE‐1146 can eradicate MM tumors established in NOD‐SCID mice. a) Comparison of TE‐1146 monotherapy with daratumumab (Dara), lenalidomide (Lena), and their combination in an H929 MM xenograft model (n = 3 to 4, treated on day 14 post‐transplantation indicated by the red star); mice were observed for 28 days post‐treatment. b) Tumors collected from experiments on the final day of observation were weighed and photographed. c) End‐point tumor weight of experiments for the different treatments. d–f) are the same as a–c) except that TE‐1146 monotherapy was compared to a high‐dose daratumumab and lenalidomide combo and n = 6; (40) stands for 40 nmol kg^−1^; (80) stands for 80 nmol kg^−1^. g–i) is the same as a–c) except that TE‐1146 monotherapy was compared to daratumumab and lenalidomide combo in an H929 MM xenograft model in the presence of human peripheral blood mononuclear cells and n = 6. *p < 0.05; **p < 0.01; ***p < 0.001. Data are shown as mean ± SEM.

The results in **Figure** **9**a–c show that, compared to the PBS control, daratumumab or lenalidomide inhibited tumor growth, with the combo treatment showing stronger inhibition. Remarkably, TE‐1146 eradicated tumors, leaving only a minuscule nodule in one mouse, despite its much lower mAb‐conjugated lenalidomide dose (20 × 6 = 1.2 × 10^2^ nmol kg^−1^) compared to cumulative lenalidomide over 28 days (46 µmol kg^−1^ day^−1^ × 28 days = 1.288 × 10^6^ nmol kg^−1^). Even with the daratumumab dose doubled/quadrupled (40/80 nmol kg^−1^) in the daratumumab/lenalidomide combo, only partial tumor growth retardation was observed in the initial two weeks (Figure 9d–f), but not thereafter. In contrast, TE‐1146 eliminated H929 xenografts. Similar results were observed for MM.1S xenografts: Compared to PBS, TE‐1146 led to the eradication or significant reduction of tumors, while daratumumab (20 nmol kg^−1^) only moderately inhibited tumor growth (Figure S11a–c, Supporting Information).

To investigate the potential immune‐activating effects, we introduced human peripheral blood mononuclear cells (PBMC) as a source of human immune cells in the immunodeficient NOD‐SCID xenograft model. Mice with H929 xenografts received intravenous injections of human PBMC along with a single TE‐1146 dose (20 nmol kg^−1^) or daratumumab (20 nmol kg^−1^) combined with lenalidomide (46 µmol kg^−1^ day^−1^). Although the daratumumab/lenalidomide combo with human PBMC showed enhanced efficacy (Figure 9g–i) compared to that without (Figure 9a–c), TE‐1146 remained significantly more effective than the combo treatment, even with human PBMC present.

### 2.11 TE‐1146 In Vivo Efficacy as a Function of its Dose

**Figure 10:**
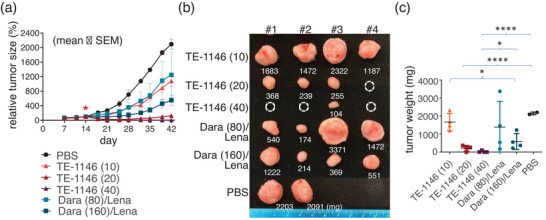
In vivo efficacy of TE‐1146 as a function of dosage in MM.1S derived xenograft mouse model. a) Comparison of TE‐1146 monotherapy to high‐dose daratumumab (Dara) and lenalidomide (Lena) combo in an MM.1S MM xenograft model (n = 2−4/group, treated on day 14 post‐transplantation). Mice were observed for 28 days post‐treatment. (10), (20), (40), (80), and (160) denote 10, 20, 40, 80, and 160 nmol kg^−1^, respectively. b) Tumors collected on the final day of observation were weighed and photographed. c) End‐point tumor weight of (b) for the different treatments. *p < 0.05; **p < 0.01; ***p < 0.001; ****p < 0.0001. Data are shown as mean ± SEM.

We evaluated how varying the TE‐1146 dose affected its effectiveness against MM tumors in MM.1S xenografts. The results in **Figure** **10** show that TE‐1146 was more efficacious in reducing MM tumors than daratumumab at higher dosages in combination with lenalidomide: TE‐1146, at 10 nmol kg^−1^, did not significantly reduce the tumor size compared to PBS. Doubling the TE‐1146 dose to 20 nmol kg^−1^ led to significant tumor reduction and eradication in one mouse. Further doubling the dose to 40 nmol kg^−1^ resulted in tumor eradication in 3 mice, with a small tumor remaining in one mouse. On the other hand, daratumumab at 80 nmol kg^−1^, combined with 46 µmol kg^−1^ day^−1^ of lenalidomide, showed growth inhibition in only two out of four mice. Doubling the daratumumab dose to 160 nmol kg^−1^ curbed tumor growth but did not eradicate the tumors in all four mice.

## 3 Discussion

Our HighDAR platform enables the construction of stable and homogenous ADCs with a higher DAR compared to most current methods. It utilizes a novel “multi‐arm linker”, incorporating a peptide core with multiple drug‐linking arms to create a drug bundle, and an N‐terminal maleimide to enable conjugation to the targeting antibody. It also employs a designed peptide containing a Zn^2+^‐binding Cys^−^ with higher reactivity toward maleimide than protonated Cys in “ordinary” peptides or engineered Cys with typical pK_a_ of ≈8.6^[^ ^26^ ^]^ and disulfide‐bonded Cys,^[^ ^27^ ^]^ enabling drug bundle conjugation to engineered Zn^2+^‐bound cysteines rather than the native disulfide‐bonded cysteines of the antibody, resulting in defined attachment sites. It also positions the drug bundles far from the antigen‐binding site to preserve the antibody structure, binding affinity, and internalization capability. The maleimide ring‐opening reaction further safeguards against premature drug bundle dissociation through exchange with endogenous thiol‐containing molecules, ensuring ADC stability until target engagement.

Our HighDAR platform offers several advantages: 1) Our “multi‐arm linker” enables the attachment of *multiple* drug molecules, leading to a higher DAR compared to linkers that attach only one drug per antibody site. 2) It renders the drug bundle water‐soluble, enabling coupling with hydrophilic antibodies in solution. In contrast, direct conjugation of hydrophobic drug molecules to antibody functional groups may expose the antibody to harsh chemical conditions that could denature it. 3) Our approach does not require enzyme‐catalyzed reactions or engineered cell lines, thereby simplifying production processes.

To illustrate the utility of the HighDAR platform, we developed TE‐1146, comprising two lenalidomide bundles conjugated to a reconfigured CD38‐specific daratumumab mAb. Consistent with our calculations showing that Zn^2+^ binds and activates each C‐terminal Cys to react with maleimide, the two lenalidomide bundles were conjugated specifically to the engineered cysteines at the C‐termini of TE‐1146. The precise conjugation yielded a homogeneous DAR‐6 ADC. Despite its smaller scFv‐IgG.Fc size and higher DAR, TE‐1146 displayed stability in plasma and in vivo with no premature release of lenalidomide.

TE‐1146 delivered the desired toxicity to tumor cells by tightly binding to CD38 on the MM cell surface. This forms a CD38/TE‐1146 complex, which is efficiently internalized via endocytosis and cleaved by lysosomal cathepsin B, releasing cytotoxic lenalidomide. This mechanism distinguishes TE‐1146 from lenalidomide and daratumumab: lenalidomide alone cannot easily enter cells, whereas daratumumab relies on Fc‐dependent effector functions to kill CD38‐expressing tumor cells. Despite carrying only ≈0.01% of the lenalidomide dosage used in the daratumumab and lenalidomide combination, TE‐1146 exhibits more potent cytotoxic effects than lenalidomide, daratumumab, or their combination in both in vitro and in vivo assays. Since TE‐1146 and daratumumab exhibit similar Fc‐mediated immune activities on CD38‐expressing MM cells, its enhanced target cell‐killing activity compared to daratumumab, alone or combined with lenalidomide, can be attributed to its efficient entry into MM cells and subsequent release of cytotoxic lenalidomide. This underscores that the efficacy of lenalidomide in eradicating MM cells hinges on its ability to enter these cells rather than on high concentrations of free lenalidomide in the bloodstream or near MM cells. Once inside MM cells, lenalidomide binds to cereblon ubiquitin ligase, altering its substrate specificity, leading to the ubiquitination and proteasomal degradation of proteins essential for MM cell survival.^[^ ^28^ ^]^

Our findings suggest that TE‐1146 holds potential as a more effective therapeutic option for hard‐to‐treat MM patients compared to the current daratumumab/lenalidomide combination. Standard MM treatment involving high doses of lenalidomide often results in severe toxicity, especially in elderly patients. Even when free lenalidomide is combined with daratumumab, only a limited lenalidomide amount can enter MM cells. Therefore, TE‐1146, by delivering lenalidomide specifically to target MM cells, offers a broader therapeutic window. As TE‐1146 is the only ADC incorporating lenalidomide in development, its therapeutic potential would be of great interest. If TE‐1146 is successful in clinical trials, the HighDAR platform, with its distinctive conjugation chemistry and novel drug bundle concept, holds promise for the development of other ADCs.

In summary, by integrating a novel “multi‐arm linker” to create drug bundles and strategically designed Cys‐containing Zn^2+^‐binding motifs fused at the C‐termini of a scFv‐IgG.Fc antibody to achieve site‐specific drug conjugation, the HighDAR platform enables the production of homogenous, stable, high‐DAR ADCs. TE‐1146, an example of this platform, is a homogenous DAR‐6 ADC that remains stable in circulation without premature payload release. It effectively targets human CD38 to concentrate lenalidomide in MM cells, producing a robust anti‐cancer effect at the tumor site.

## 4 Experimental Section

### Animals

All animal experiments strictly adhered to internationally accepted ethical principles for the care and use of laboratory animals (IACUC: BioTReC‐110‐D‐031‐R1). Male C57BL/6 mice, aged 6 weeks, were purchased from Taiwan's National Laboratory Animal Center. Additionally, male and female NOD.CB17‐Prkdcscid/NcrCrlBltw mice, also 6 weeks old, were purchased from the Institute of Cellular and Organismic Biology Laboratory Animal Facility of Academia Sinica, Taiwan. The mice were housed in groups of 3–5 in a well‐ventilated, humidified room with a 10 h dark to 14 h light cycle. They had unrestricted access to water and were fed a standard chow diet. Before starting experiments, all mice were accustomed to blood sampling and dosing procedures for one week to minimize stress due to handling.

### Cell Culture

Human MM cell lines, H929 (CRL‐9068), MM.1S (CRL‐2974), and U266 (TIB‐196), and Burkitt's lymphoma cell line Daudi (CCL‐213), were purchased from ATCC (American Type Culture Collection). All these cells were cultured in RPMI 1640 (Gibco) supplemented with 10% fetal bovine serum (Gibco) and antibiotics (100 U mL^−1^ penicillin and 100 µg mL^−1^ streptomycin, Corning). The cultures were maintained at 37 °C in a well‐regulated (5% (v/v)) CO_2_ atmosphere.

### Constructing a Zn^2+^‐Binding Motif

First, we searched the Protein Data Bank (PDB)^[^ ^29^ ^]^ for structures containing Zn^2+^‐binding CXXH motifs, where X represents small residues such as Ala, Gly, and Pro. In devising the composition of the Zn^2+^‐binding CXXH motif, we excluded large hydrophobic residues that would reduce solubility and polar residues (e.g., Asn/Gln or Ser/Thr) that may form hydrogen bonds with the Zn^2+^‐bound Cys, thereby decreasing its reactivity, as shown in previous studies.^[^ ^30^ ^]^ In several PDB structures (e.g., PDB 3qqc), we identified a **C** PG **H** motif where the Cys and His are bound to Zn^2+^, which is also bound by two other cysteines upstream forming a tetrahedral Zn^2+^‐CCCH site with a well‐conserved conformation (Figure S12a, Supporting Information). A Zn^2+^‐binding motif for site‐specific conjugation to the drug bundle was derived from this Zn^2+^‐CCCH conformation by i) replacing the first two Cys with water molecules, ii) adding an Ala to the **C** PG **H** motif, and iii) acetylating the N‐ and C‐termini to yield a Zn^2+^‐CHww site with a net charge of +1 (Figure S12b, Supporting Information).

### Geometry Optimization

The M06‐2X functional combined with the SDD basis set, which employs Dunning/Huzinaga full double zeta (D95)^[^ ^31^ ^]^ for H, C, N, O, and S atoms and Stuttgart/Dresden effective core potential^[^ ^32^ ^]^ for Zn^2+^, has proven to be efficient at reproducing Zn−ligand distances.^[^ ^33^ ^]^ Thus, it was used to optimize each construct using the polarizable continuum model with a dielectric constant of 78.4.^[^ ^34^ ^]^ To verify that the fully optimized structures had no imaginary frequencies, we computed M06‐2X/SDD frequencies. All calculations were performed with the Gaussian09 program^[^ ^35^ ^]^ using the default integration grid and a convergence threshold of 10^−6^.

### Computing Solution Free Energies

Since two A **C** PG **H** peptides are attached to the mAb C‐termini, a Zn^2+^ ion can bind to each A **C** PG **H** peptide to form two Zn^2+^‐CHww sites or it can bind both A **C** PG **H** peptides to form a single Zn^2+^‐CCHH site. To determine which type of Zn^2+^‐site is favored, we computed the solution free energy *Δ*G^sln^ for forming a Zn^2+^‐CCHH site from two Zn^2+^‐CHww sites in water at a temperature *T* of 298K according to:

(1) $$\Delta G^{\mathrm{sln}}∼\Delta E_{\mathrm{elec}}^{\;\;\mathrm{sln}}+\Delta E_{\mathrm{therm}}^{\;\;\mathrm{sln}}-T\Delta S^{\mathrm{sln}}$$

The thermal energy (*Δ*E_therm_^sln^) and entropy (*Δ*S^sln^) in aqueous solution were computed using the M06‐2X/SDD frequencies. The electronic energies *Δ*E_elec_^sln^ were corrected by single‐point energy calculations using M11/6‐31+G(3d,p)^[^ ^33^ ^]^ and the SMD solvation model.^[^ ^36^ ^]^

### Estimating the Reaction Barrier

To determine if Zn^2+^‐bound Cys reacts with maleimide faster than free or disulfide‐bonded Cys, we estimated the free energy barrier as follows: First, we placed an N‐methyl‐maleimide (Mal) near the Zn^2+^‐bound Cys to form various hydrogen bonds, generating 5 distinct Zn^2+^−ACPGH—Mal conformations, as shown in Figure S13 (Supporting Information). Each Zn^2+^−CHww—Mal construct was fully optimized using the M06‐2X/SDD method. Next, the distance between the Cys(S^−^) and the closest Mal carbon atom in each fully optimized structure (3.3−4 Å) was successively reduced by 0.05 Å until it reached 2 Å when an S−C bond was formed. Each intermediary structure corresponding to a fixed S—C distance was re‐optimized and the corresponding solution free energy, ΔG^sln^, was computed using eq 1. The ΔG^sln^ values of the ≈40 constrained‐optimized structures for each Zn^2+^−CHww—Mal conformation were plotted as a function of the S—C distance, as shown in Figure S14 (Supporting Information). In computing the ΔG^sln^ profile, sudden energy drops were occasionally observed, typically due to hydrogen‐bond formation or side‐chain reorientation. In such cases, the intermediary minimum was re‐optimized without constraints and used as the starting point to recompute the free energy profile. The peak of the solution free energy profile gives an estimate of the reaction barrier. The reaction barriers for the different Zn^2+^−CHww—Mal conformations were then averaged according to the Boltzmann probability of each conformation. Similarly, the free energy barriers for protonated Cys and disulfide‐bonded Cys to form an S−C bond with maleimide were estimated from the corresponding solution free energy profiles.

To ensure that the reaction barrier estimated from the peak of the ΔG^sln^ profile is close to that determined by the transition state of the reaction, the intermediary structure corresponding to the peak of the ΔG^sln^ profile was fully optimized without any constraints to locate the transition state, which was verified by vibrational frequency and intrinsic reaction coordinate calculations. For the ‘w+NH’ conformation, a transition state with only one imaginary frequency involving the carbon and sulfur atoms was found. Its free energy (11.6 kcal) was ≈1 kcal mol^−1^ lower than that estimated from the peak of the solution free energy profile. Hence, reaction barriers estimated from free energy profiles in solution can reliably indicate the relative reactivities of Zn^2+^‐bound, free, and disulfide‐bonded Cys.

### Constructing an α‐CD38 mAb with C‐Terminal Zn^2+^‐Binding Motifs

We fused the gene sequence encoding the scFv specific for human CD38, derived from daratumumab, upstream of the gene sequence encoding the IgG1‐Fc hinge region and the CH2 domain. Additionally, we fused the gene sequence encoding the Zn^2+^‐binding motif downstream of the gene sequence encoding the IgG1‐Fc CH3 domain (lacking the C‐terminal Lys) with a short (Gly)_3_ linker. The resulting gene sequence construct was placed in the Freedom pCHO 1.0 expression cassette (Thermo Fisher Scientific). The translated product is an α‐CD38 fusion protein, consisting of a dimer of (scFv α‐CD38)‐CH2‐CH3‐(Gly)_3_‐ACPGHA.

To stably express the fusion protein, the expression plasmid was linearized using Ssp*I* digestion and then transfected into CHO‐S cells using the Freedom CHO‐S Kit (Thermo Fisher Scientific). The transfected cells were incubated at 37 °C with orbital shaking (150 rpm) for 40–48 h post‐transfection. Subsequently, transfected CHO‐S cells were cultured in the presence of 10 µg mL^−1^ puromycin and 200 nM MTX for stable pool selection. The selection medium was changed twice weekly and α‐CD38 mAb expression was monitored weekly. After 2–3 weeks, suitable stable cells for α‐CD38 mAb production were cryopreserved. α‐CD38‐CHO‐S cells were cultured following the Freedom CHO‐S kit instructions. A culture supernatant was collected, and the antibody was purified using protein A sepharose (Cytiva) chromatography. After buffer exchange to PBS, the fusion protein was analyzed using 8% SDS‐PAGE, and stored at −20 °C in PBS with 50% glycerol.

### Conjugating Drug Bundles to a α‐CD38 mAb

The production of the lenalidomide drug bundle using the multi‐arm linker was outsourced to WuXi AppTec Co., Ltd. (Shanghai, China); the synthesis steps are described in the Figures S15–S17 (Supporting Information). Before drug conjugation, the purified α‐CD38 mAb was exchanged into a conjugation buffer (10 mM sodium succinate, 30 mM sucrose, pH 6.0) with a final concentration of 15 µM. It was then reduced by incubation with six equivalents of TCEP (Sigma) at 37 °C for 30 min to free the Zn^2+^‐binding Cys from any unwanted disulfide bonds with Cys or glutathione in the medium. The reduced protein was dialyzed against the conjugation buffer containing 60 µM ZnCl_2_ at 25 °C for 2 h using a slide‐A‐lyzer dialysis cassette G2 (Thermo Fisher Scientific). This served to reconstitute hinge disulfide bonds and enable Zn^2+^ to bind and activate the C‐terminal Cys. Three equivalents of lenalidomide drug bundles with a final concentration of 45 µM in a buffer (10 mM sodium succinate, 30 mM sucrose, 60 µM ZnCl_2_, pH 6) were added to the α‐CD38 mAb. The mixture was incubated in a round‐bottomed flask under stirring (600 rpm) at 25 °C for 10 min. To solubilize the products, an equal volume of 100% (w/v) sucrose was added and stirred (600 rpm) at 25 °C for 16−18 h. Thiol‐maleimide conjugates were hydrolyzed by adding 1/10 volume of the hydrolysis buffer (100 mM Tris, 100 mM NaCl, 100 mM L‐Arg, and 50% sucrose (w/v), pH 9.0) and stirring the resulting solution at 25 °C for 2 days.

### Acquiring the Final Product, TE‐1146

To remove unconjugated or single drug bundle conjugates, all conjugates were exchanged to buffer A (50 mM Na_2_HPO_4_, 1 M NaCl, pH 7.0) and applied to a pre‐equilibrated HIC, HiScreen Phenyl HP (GE Healthcare). Three washing steps were performed with 0, 50, and 65% of buffer B (50 mM sodium phosphate, pH 7) for 10, 20, and 15 column volumes, respectively. Conjugates with 2 drug bundles were eluted with 100% buffer B for 20 column volumes at a flow rate of 1.0 mL min^−1^. The collected samples were then applied to a HiPrep 26/60 Sephacryl S‐200 HR SEC column (GE Healthcare) to separate the molecular construct from protein aggregates. After HIC and SEC purifications, TE‐1146 was obtained and its purity was determined by SDS‐PAGE, HIC‐HPLC, and SEC‐HPLC. The intensity of each band separated by SDS‐PAGE was quantified using the ImageJ software (National Institutes of Health). 10 µg of purified TE‐1146 was analyzed by SEC‐HPLC (XBridge Premier Protein SEC Column, 250 Å, 2.5 µm, 4.6×150 mm, Waters) with 2× PBS as the mobile phase at a flow rate of 0.2 mL min^−1^ for 18 min. 50 µg of purified TE‐1146 was analyzed by HIC‐HPLC (Protein‐Pak Hi Res HIC, 2.5 µm, 4.6×100 mm, Waters) with gradient elution (A: 1.25 M ammonium sulfate with 25 mM sodium phosphate, pH 7.0; B: 25 mM sodium phosphate, pH 7.0) at a flow rate of 0.6 mL min^−1^ for 35 min.

### Molecular Weight (MW) Analysis of TE‐1146

The purified TE‐1146 was further analyzed using 2 techniques: 8% non‐reducing SDS‐PAGE and matrix‐assisted laser desorption/ionization time‐of‐flight (MALDI‐TOF) by the Mass Core Facility of the Institute of Molecular Biology at Academia Sinica, Taiwan. For MALDI‐TOF, purified TE‐1146 was directly spotted onto a MALDI target plate using saturated sinapinic acid (2 mg mL^−1^ in 0.1% trifluoroacetic acid (TFA) in 30:70 acetonitrile:water, v/v). Each spot was analyzed using a Bruker Autoflex III MALDI TOF/TOF (Bremen, Germany), equipped with a 200 Hz SmartBeam Laser. Data acquisition and processing were made by FlexControl 3.4 and Flex‐Analysis 3.4 (Bruker Daltonik GmbH).

### Analyzing Drug Conjugation Sites on TE‐1146

To reduce the disulfide bonds, TE‐1146 (2 µg) was dissolved in PBS at pH 7.4 and incubated with 18 mM TCEP in 17 mM triethylammonium bicarbonate buffer at 55 °C for 10 min, away from light. The reduced protein was incubated with 15.6 mM iodoacetamide in the dark at room temperature for 30 min and subsequently incubated with trypsin (0.2 µg) at 37 °C overnight. The samples were detected by LC‐ESI‐MS on an Orbitrap Fusion mass spectrometer equipped with EASY‐nLC 1200 system (Thermo Fisher Scientific). A 5 µl digested solution was injected into an Easy‐Column capillary column (C18, 0.075×150 mm, ID 3 µm) at a flow rate of 1 µL min^−1^. Chromatographic separation utilized a flow rate of 300 nL min^−1^ with 100% mobile phase A (0.1% formic acid in water), which was reduced to 98% by 2% mobile phase B (0.1% formic acid in 80% acetonitrile) at 2 min, and further reduced to 60% by 40% mobile phase B at 40 min. Full‐scan mass spectrometry was recorded, and the target m/z was isolated for collision‐induced dissociation with a normalized collision energy of 35 and a maximum injection time of 100 ms. LC‐MS spectra were searched for expected molecular weights of peptides with an additional MW of a carbamidomethyl group (58 Da) or a lenalidomide bundle (4207.2 Da). The Mascot search engine was used to identify cysteine‐containing peptide sequences.

### Generating Stable Human CD38 HEK293T Cells

The full‐length human CD38 cDNA ORF clone was purchased from Sino Biological Inc. and cloned into a pCDH‐CMV‐MCS‐Ef1α‐Puro expression vector, generating the lentiviral‐based *CD38*‐expressing plasmid, pCDH‐CMV‐CD38. Lentivirus packaging involved co‐transfecting pCDH‐CMV‐CD38, pCMV‐VSV‐G, and pCMV‐δR8.91 into HEK293T cells. Following lentivirus infection, human CD38‐overexpressing HEK293T cells were cultured with 1 µg mL^−1^ puromycin three days post‐infection. Flow cytometry confirmed the successful overexpression of human CD38.

### Flow Cytometry Cell‐binding Assay

The binding of TE‐1146 and daratumumab to various cell lines in vitro was assessed using flow cytometry on ice. About 2 × 10^5^ of H929, MM.1S, U266, or Daudi cells were washed thrice with flow cytometry staining (FACS) buffer (PBS with 1% fetal bovine serum) and incubated with different concentrations of TE‐1146 and daratumumab (1 µM, 100, 10, and 1 nM, 100, 10 and 1 pM) for 20 min. After FACS buffer rinsing, cells were labeled with PE‐conjugated anti‐human IgG‐Fc antibody (Jackson ImmunoResearch) to detect the presence of TE‐1146 and daratumumab on the cell surface. Unstained cells and cells with secondary antibodies only were used as negative controls. FACSCanto II with FlowJo software (BD Bioscience) measured cell‐associated fluorescence.

### Plasma Stability Test

The stability of α‐CD38 mAb, TE‐1146, or daratumumab was assessed by dissolving them in human plasma to achieve a final concentration of 2 mg mL^−1^, followed by incubation at 37 °C for 28 days. ELISA analysis at specific intervals (days 0, 3, 7, 10, 14, 21, and 28) involved coating recombinant human CD38 proteins to capture daratumumab, α‐CD38 mAb, and TE‐1146 (see Figure 4a). The three CD38‐binding antibodies were detected by a secondary antibody, HRP‐conjugated anti‐human IgG‐Fc antibody (Jackson ImmunoResearch). To ascertain the presence of the two lenalidomide bundles on TE‐1146, an anti‐lenalidomide‐bundle scFv‐mouse IgG‐Fc antibody (screened by the human therapeutic antibody development platform core facility of Biomedical Translation Research Center, Taiwan), was used, and detected by an HRP‐anti‐mouse IgG‐Fc antibody (Jackson ImmunoResearch).

A 96‐well ELISA plate was coated with recombinant human CD38 protein (0.1 µg 100 µL^−1^ per well) for 16 h at 4 °C. Subsequently, wells were washed with PBST buffer (PBS with 0.1% Tween‐20), and blocked with a blocking buffer (PBS with 1% bovine serum albumin) for 30 min. Standards were prepared by dissolving TE‐1146 with human plasma and diluting it in the blocking buffer. All samples were diluted in the blocking buffer to match the linearity of the calculation curve. The standards and diluted samples were added to each well and incubated at room temperature for 1.5 h. Following three washes with PBST, the secondary antibodies with proper dilution in the blocking buffer were added to the corresponding well. To detect drug‐conjugated TE‐1146, the HRP‐anti‐mouse IgG‐Fc antibody was introduced after washing off the anti‐lenalidomide‐bundle scFv‐mouse IgG‐Fc antibody. Finally, the concentrations of each antibody were quantified using a tetramethylbenzidine reagent (ScyTeK) and a microplate reader (Synergy H1, BioTek). The half‐life (T_1/2_) was calculated by the PKSolver software.

### Pharmacokinetics in Mouse

Male C57BL/6J wild‐type mice were intravenously injected with 60 µg 0.02 kg^−1^ of TE‐1146 or 80 µg 0.02 kg^−1^ of daratumumab. Blood samples from the orbital sinus were collected at 2 h and on days 1, 2, 4, 7, 10, 14, 21, and 28. Plasma, obtained through centrifugation, was stored at −80 °C until analysis. The concentrations of TE‐1146 or daratumumab in plasma were determined by ELISA, using the same procedure as in the plasma stability test. Pharmacokinetic parameters were calculated using the IV‐bolus two‐compartmental method with PKSolver software.

### In Vivo Tumor Targeting

To establish a subcutaneous xenograft tumor model, 2 × 10^7^ H929 cells in PBS, mixed with 50% (v/v) extracellular matrix gel (Matrigel, Corning), were subcutaneously injected into the flanks of NOD‐SCID mice (The Jackson Laboratory). TE‐1146 was labeled with DyLight 680 NHS‐Ester (Thermo Fisher Scientific) at a dye‐to‐protein ratio of 2.3. The binding ability of DyLight 680‐labeled TE‐1146 to CD38 was determined by a cell‐based ELISA. Human CD38‐overexpressing HEK293T cells, seeded at 100 000 cells per well in 96‐culture plates overnight, were fixed at 4 °C for 30 min using 100% ethanol. Subsequent steps, including washing, sample addition, and signal detection, followed the protocol outlined in the “Plasma Stability Test” section. Mice bearing H929 tumors were injected intraperitoneally with 60 µg 0.02 kg^−1^ DyLight 680‐labeled TE‐1146. Non‐invasive whole‐body imaging for tumor targeting assessment was performed using an IVIS 100 Imaging system (Xenogen, Alameda, CA). Images were acquired at 2, 4, 24, 48, and 120 h post‐injection.

### Cellular Internalization Assay

The cellular internalization efficiency of TE‐1146 in vitro was evaluated using flow cytometry and confocal microscopy analyses.

### Flow Cytometry

For the flow cytometry assay, 2 × 10^5^ H929 cells per well were incubated with 1 µM TE‐1146 in FACS buffer for 20 min on ice. The cells were washed and resuspended in fresh medium, then incubated at 37 °C for 0.5, 1, 2, and 3 h. After washing, the cells were incubated with PE‐conjugated goat anti‐human IgG‐Fc antibody for 20 min on ice. After washing, the cell‐associated fluorescence was determined using a FACSCanto II flow cytometer with analysis carried out using the FlowJo software. The internalization percentage was calculated from δM, the mean fluorescence intensity at a given time *t* relative to the background mean fluorescence intensity, according to:

(2) $$\%\;\mathrm{internalization}=\left[\delta M_{\left(t=0\right)}-\delta M_{\left(t=x\right)}\right]\times 100/\delta M_{\left(t=0\right)}$$

### Confocal Microscopy

Before confocal microscopy analysis, TE‐1146 was labeled with a fluorescent dye, VivoTag 645 (PerkinElmer), at a dye‐to‐protein ratio was 2.3, following the provided kit's standard protocol, and its binding affinity was evaluated by cell‐based ELISA. 1 × 10^7^ H929 cells were stained with 100 nM TE‐1146‐VivoTag 645 at 4 °C for 30 min, followed by replacement with pre‐warmed fresh medium and transfer to a 37 °C incubator. At 0, 1, 2, and 3 h, 1 × 10^6^ cells were collected, fixed with 100% ethanol at room temperature for 20 min, and prepared slides with Fluoroshield Mounting Medium containing 4′,6‐diamidino‐2‐phenylindole (DAPI) (Abcam) to label cell nuclei. The slides were then analyzed by confocal microscopy (Zeiss LSM880 Airyscan).

### Drug‐Releasing Assay

Lenalidomide (1 µM) cultured in PBS or RPMI medium, TE‐1146 (1 µM) in RPMI medium, H929 cells in RPMI medium, and TE‐1146 (1 µM) co‐cultured with 1 × 10^6^ H929 cells were incubated at 37 °C for 5 days. After incubation, supernatants were collected and subjected to centrifugation to remove any cellular debris. Molecules with MW < 3 kDa were concentrated using the Amicon Ultra‐0.5 (3 kDa, Merck Millipore) filtration system. Subsequently, the prepared samples were injected into a Hypersil GOLD C8 HPLC Column (Thermo Fisher Scientific) for HPLC analysis. The HPLC system was operated in gradient mode with a flow rate of 1 mL min^−1^. The HPLC program commenced with 0% solvent B (consisting of acetonitrile with 0.1% TFA) and maintained for 8 min. Then a linear gradient was applied, increasing from 0% to 100% solvent B within 20 min. After a 10 min retention of pure solvent B, the column was regenerated with 100% solvent A (comprising water with 0.1% TFA) within 7 min. The injection volume was 100 µL, the column oven temperature was 25 °C, and the total run time was 45 min.

### In Vitro Cytotoxicity Assessment

To evaluate the cytotoxic effects of various drugs, H929, U266‐CD38^−^, MM.1S, and Daudi cells were seeded at 5000 cells per well in 96‐well plates and cocultured with fresh medium containing different drug concentrations for varying durations (5 h, 1 day, 3 or 5 days) at 37 °C. Cell viability was determined using the alamarBlue cell viability reagent (Thermo Fisher Scientific). About 10 µL alamarBlue cell viability reagent was added to achieve a final concentration of 10% v/v and incubated at 37 °C for 1.5 h. The fluorescence was measured on a microplate reader in arbitrary fluorescent units following excitation at 560 nm and emission at 590 nm.

### Human Peripheral Blood Mononuclear Cells (PBMC) Preparation

Human whole blood units were obtained from healthy donors through Access Biologicals (CA, USA) with Institutional Review Board‐approved informed consent (approval by Advarra, Inc.; CR00277366). PBMCs were isolated from whole blood using Ficoll‐Hypaque density‐gradient centrifugation. They were then used as effector cells in the ADCC and CDC assays as well as in the in vivo mouse tumor model.

### ADCC Analysis

To assess ADCC, an LDH cytotoxicity assay kit (Enzo) was used to detect the amount of LDH released from target cells that were killed by the tested drug and effector cells (freshly isolated PBMC). The target cells (H929, MM.1S, U266‐CD38^+^, U266‐CD38^−^, and Daudi cells) were seeded at a density of 5000 cells per well in a 96‐well plate. Control human IgG (at 7 concentrations ranging from 500−0.032 nM with serial 5‐fold dilutions), TE‐1146, daratumumab, lenalidomide at 6 times the molar amounts of the antibodies (at 7 concentrations ranging from 3000−0.192 nM with serial 5‐fold dilutions), or daratumumab/lenalidomide combinations at the respective concentrations were co‐cultured with target and effector cells (1 × 10^5^ cells per well, effector:target ratio of 20:1) at 37 °C. After incubation for 5 or 24 h, supernatants were collected and analyzed by LDH cytotoxicity assay kit.

### CDC Analysis

This mirrored the ADCC analysis using the LDH cytotoxicity assay kit. Target cells and drugs/control were the same as in the ADCC experiments, with 10% human plasma serving as the complement protein source. Cytolysis of the target cells was determined after a 5 h incubation.

### ADCP Analysis

eGFP‐overexpressing H929 cells (H929‐eGFP) were transduced with lentivirus infection and cultured with 10 µg mL^−1^ puromycin three days post‐infection. Figure S10 (Supporting Information) illustrates the procedure to determine the ADCP activity of TE‐1146. Flow cytometry confirmed the eGFP expression in H929‐eGFP cells. H929‐eGFP, THP‐1 cells (ATCC), and different concentrations of TE‐1146, daratumumab, or control IgG were mixed and cultured for 5 h at 37 °C. After incubation, cells were stained with allophycocyanin (APC)‐conjugated anti‐human CD13 antibody (BioLegend) and assessed by flow cytometry. The proportion of double‐positive cells displaying both eGFP and APC signals represented the ADCP efficacy.

### In Vivo Tumor Reduction Assay in Mice

To generate subcutaneous tumors, 2 × 10^7^ H929 cells in PBS, along with 50% (v/v) extracellular matrix gel, were subcutaneously injected into the flanks of NOD‐SCID mice. Tumor size and body weight were recorded every 2 to 3 days. On day 14 post‐transplantation, mice with tumors within 150 ± 20 mm^3^ range were selected for anti‐tumor efficacy tests. Tumor volumes, measured using an external caliper, were calculated using the modified ellipsoid formula: (length × width × width)/2.

TE‐1146 and daratumumab, prepared in PBS, were administered intraperitoneally. The TE‐1146 dose was 10, 20, or 40 nmol kg^−1^, whereas the daratumumab dose was 20, 40, 80, or 160 nmol kg^−1^. Lenalidomide (46 µmol kg^−1^) was injected intraperitoneally daily. At the end of the experiments, tumors were collected and weighed. To study the effect of human PBMC in vivo, 1 × 10^7^ human PBMCs per mouse were intravenously injected 0.5 h before the administration of different drugs on day 14 post‐implantation.

### Statistical Analysis

Data analysis employed GraphPad Prism V9.0 software. Results were reported as mean ± SD or mean ± SEM as indicated in the figures. Statistical significance between groups was assessed using the Student t‐test, whereas statistical differences among multiple groups were analyzed by one‐way ANOVA with Bonferroni's multiple comparison post‐tests.

## Conflict of Interest

T. W. Chang is the founder and current director of Immunwork, Inc. All authors except C. Lim are employees of Immunwork, Inc. All authors hold stock or stock options from Immunwork, Inc.

## Author Contributions

T.‐W.C. conceived the platform rationale and designed the multi‐arm linker. C.G., H.‐M.C., C.L., and T.‐W.C. designed the Zn^2+^‐binding motif for site‐specific conjugation. Y.‐H.Y. designed and performed in vitro and in vivo experiments. M.‐Y.H. and P.‐H.L. assist in vivo experiments. Y.‐H.Y. and T.‐W.C. analyzed and interpreted data. W.‐T.T., W.‐C.L., and P.‐W.W. constructed and characterized TE‐1146. H.‐J.L. produced mAb and CD38‐overexpressing cells. C.L., Y.‐H.Y., and T.‐W.C. wrote the paper and C.‐J.P. wrote “Synthesis of lenalidomide drug bundle” in Supporting Information.

## Supporting information

Supporting Information

## Data Availability

The data that support the findings of this study are available from the corresponding author upon reasonable request.
