# Supplementary material for: An Antibody‐Drug Conjugate for Multiple Myeloma Prepared by Multi‐Arm Linkers
Source: Adv Sci (Weinh). 2024 Mar 13;11(20):2307852. doi: 10.1002/advs.202307852 (PMC11132082; doi:10.1002/advs.202307852)
Supplement: Supplementary file 1 — Supporting Information [file ADVS-11-2307852-s001.pdf]

ADVANCED SCIENCE
Open Access

Supporting Information

for *Adv. Sci.*, DOI 10.1002/advs.202307852

An Antibody-Drug Conjugate for Multiple Myeloma Prepared by Multi-Arm Linkers

Yueh-Hsiang Yu, Wei-Ting Tian, Cédric Grauffel, Wei-Chen Lin, Ming-Yu Hsieh, Pei-Wen Wu, Hui-Ju Lee, Chi-Jiun Peng, Pei-Hsuan Lin, Hsing-Mao Chu, Carmay Lim and Tse Wen Chang**

Supplementary Information

An Antibody-Drug Conjugate for Multiple Myeloma Prepared by Multi-arm Linkers

Yueh-Hsiang Yu,[†]* Wei-Ting Tian,[†] Cédric Grauffel,[†] Wei-Chen Lin,[†] Ming-Yu Hsieh,[†] Pei-Wen Wu,[†] Hui-Ju Lee,[†] Chi-Jiun Peng,[†] Pei-Hsuan Lin,[†] Hsing-Mao Chu,[†] Carmay Lim,[#]* and Tse Wen Chang,[†]*

[†]Immunwork, Inc., Academia Rd., Sec. 1, Nangang, Taipei 11571, Taiwan

[#]Institute of Biomedical Sciences, Academia Sinica, Academia Rd., Taipei 115, Taiwan

(a)

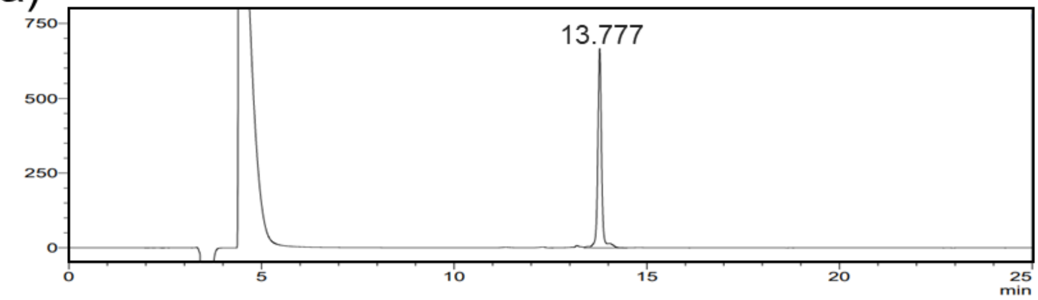

Peak Results (Area Percent at least 1%)			
RT (min)	Height	Area	Area%
13.777	634185	4553633	100

(b)

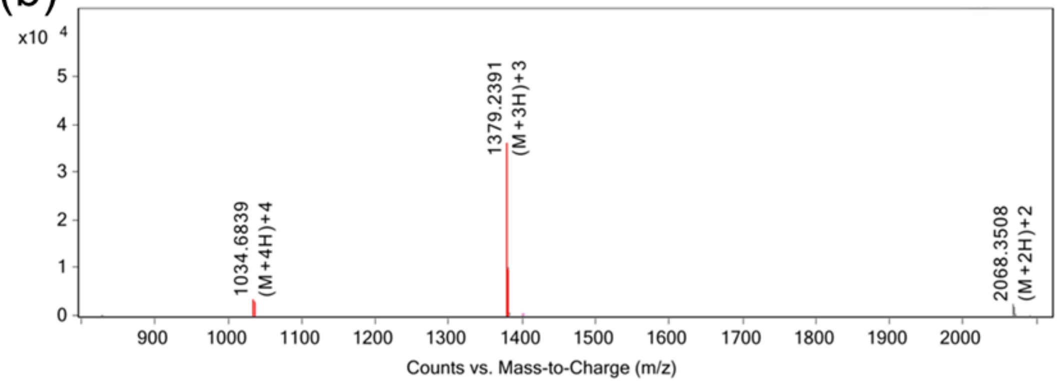

Supplementary Figure S1. **The molecular weight of lenalidomide drug bundle.** (a) The HPLC and (b) ESI-MS results of lenalidomide bundle.

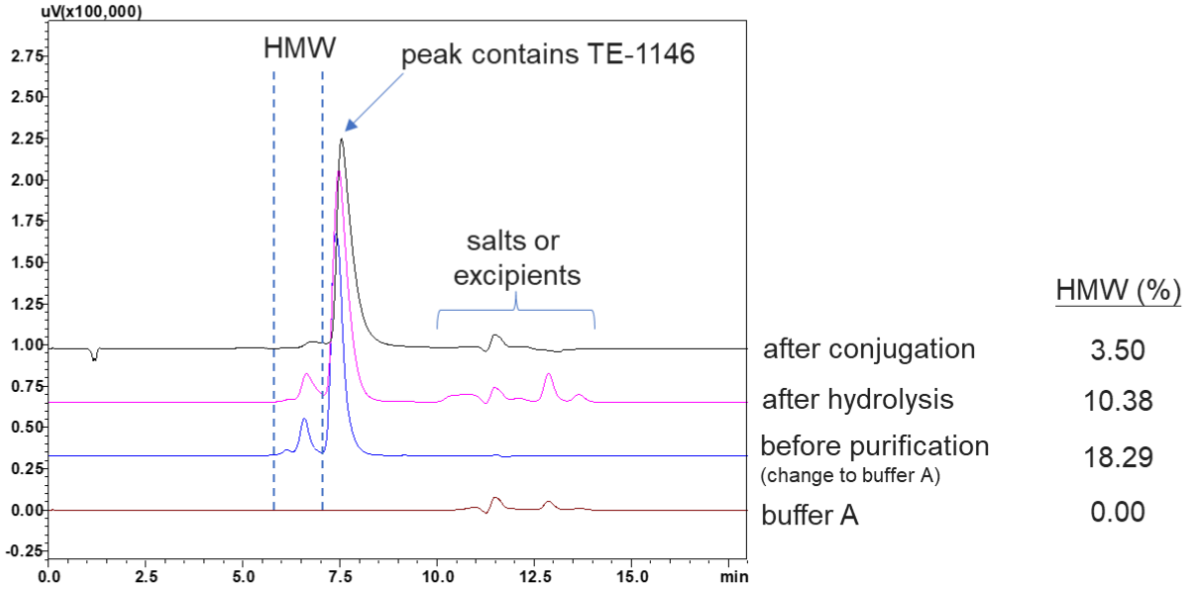

Supplementary Figure S2. The percentage of high molecular weight aggregation before purification. Samples collected from three different steps during TE-1146 preparation were analyzed by SEC-HPLC. The peak area of high MW was gated (marked by HMW) and calculated. The peak area percentages are listed on the right.

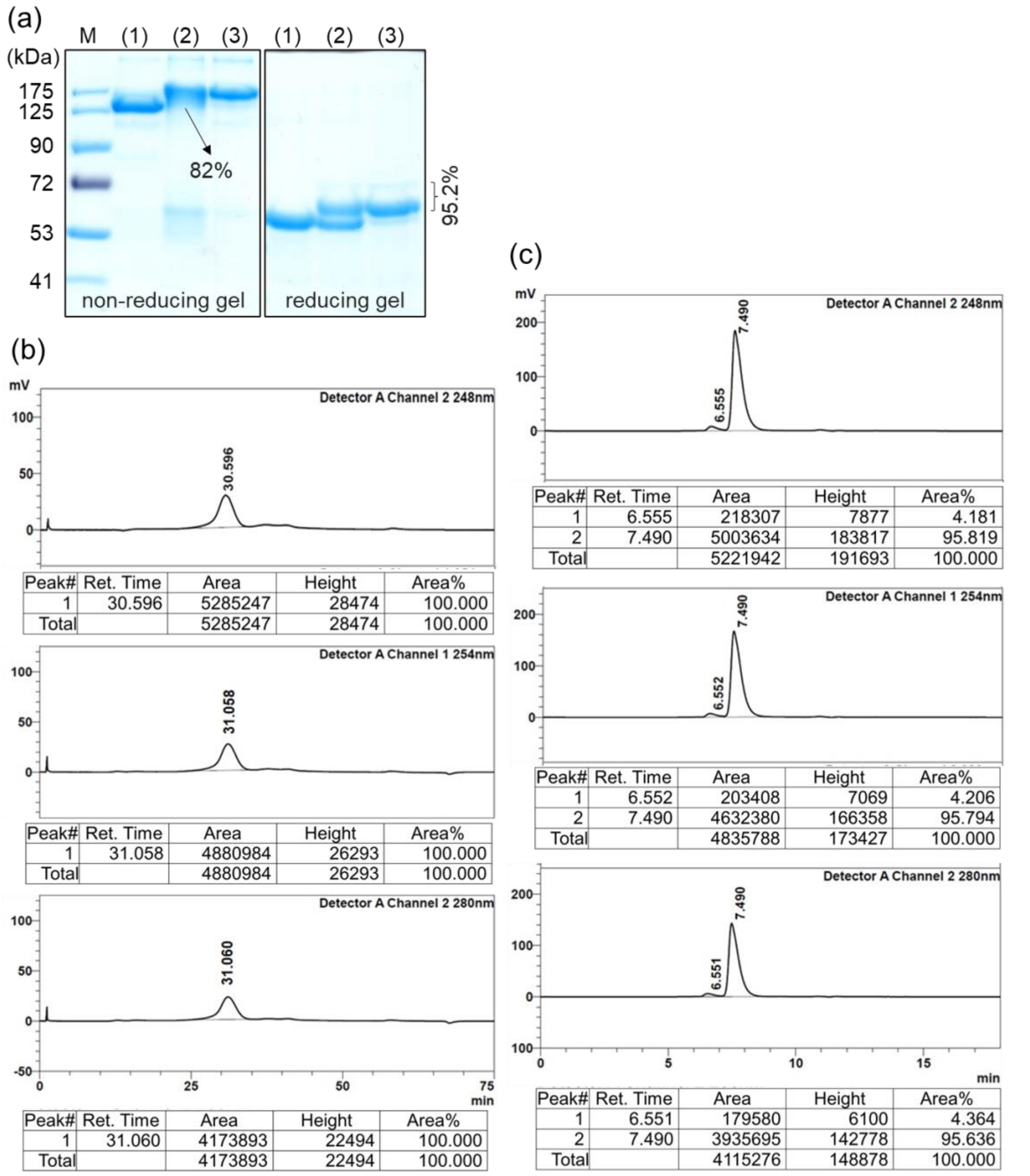

Peak#	Ret. Time	Area	Height	Area%
1	30.596	5285247	28474	100.000
Total		5285247	28474	100.000

Peak#	Ret. Time	Area	Height	Area%
1	31.058	4880984	26293	100.000
Total		4880984	26293	100.000

Peak#	Ret. Time	Area	Height	Area%
1	31.060	4173893	22494	100.000
Total		4173893	22494	100.000

Peak#	Ret. Time	Area	Height	Area%
1	6.555	218307	7877	4.181
2	7.490	5003634	183817	95.819
Total		5221942	191693	100.000

Peak#	Ret. Time	Area	Height	Area%
1	6.552	203408	7069	4.206
2	7.490	4632380	166358	95.794
Total		4835788	173427	100.000

Peak#	Ret. Time	Area	Height	Area%
1	6.551	179580	6100	4.364
2	7.490	3935695	142778	95.636
Total		4115276	148878	100.000

Supplementary Figure S3. **Purity of TE-1146**. (a) The molecular weight discrepancy of unconjugated α-CD38 mAb (1), the intact products right after conjugation process (2), and purified TE-1146 (3) were displayed by SDS-PAGE analysis with non-reducing gel (left) and reducing gel (right). The conjugation yield of the intact products right after conjugation process is over 80% identified by ImageJ (National Institutes of Health) software. The purity identified by ImageJ is approximately 95%. M stands for protein marker. (b) The HIC-HPLC and (c) SEC-HPLC profile of the purified TE-1146 (top: 248 nm; middle: 254 nm; bottom: 280 nm). The profile displays high purity of TE-1146 (> 95%) after HIC and SEC purification.

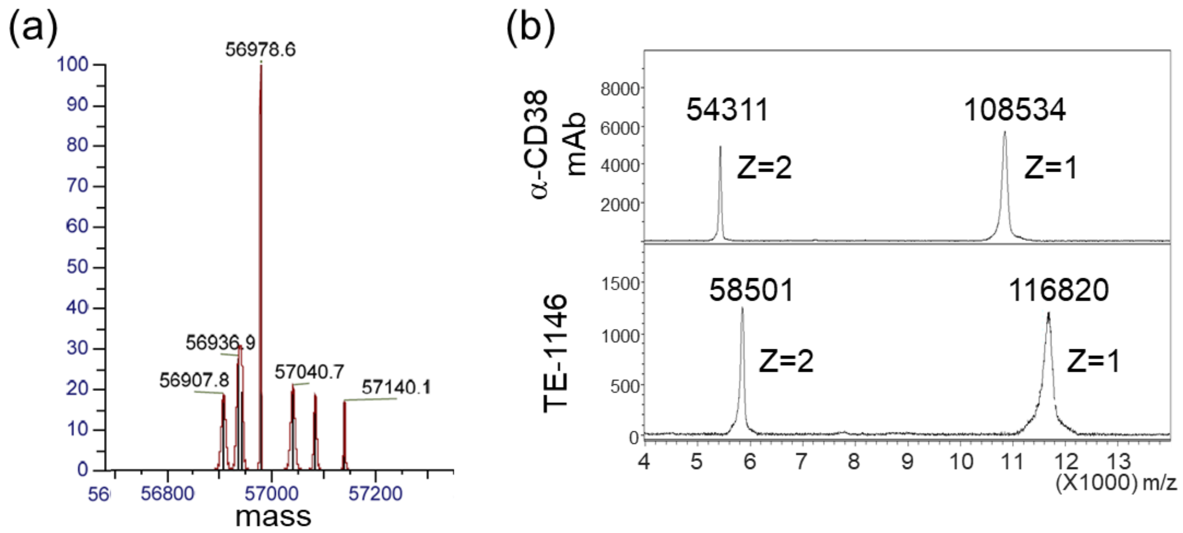

Supplementary Figure S4. DAR of TE-1146 from MW analysis. (a) The exact MW of reduced TE-1146 determined by LC-MS analysis. (b) The molecular weights of α-CD38 mAb (top panel) and purified TE-1146 (bottom panel) detected by MALDI-TOF.

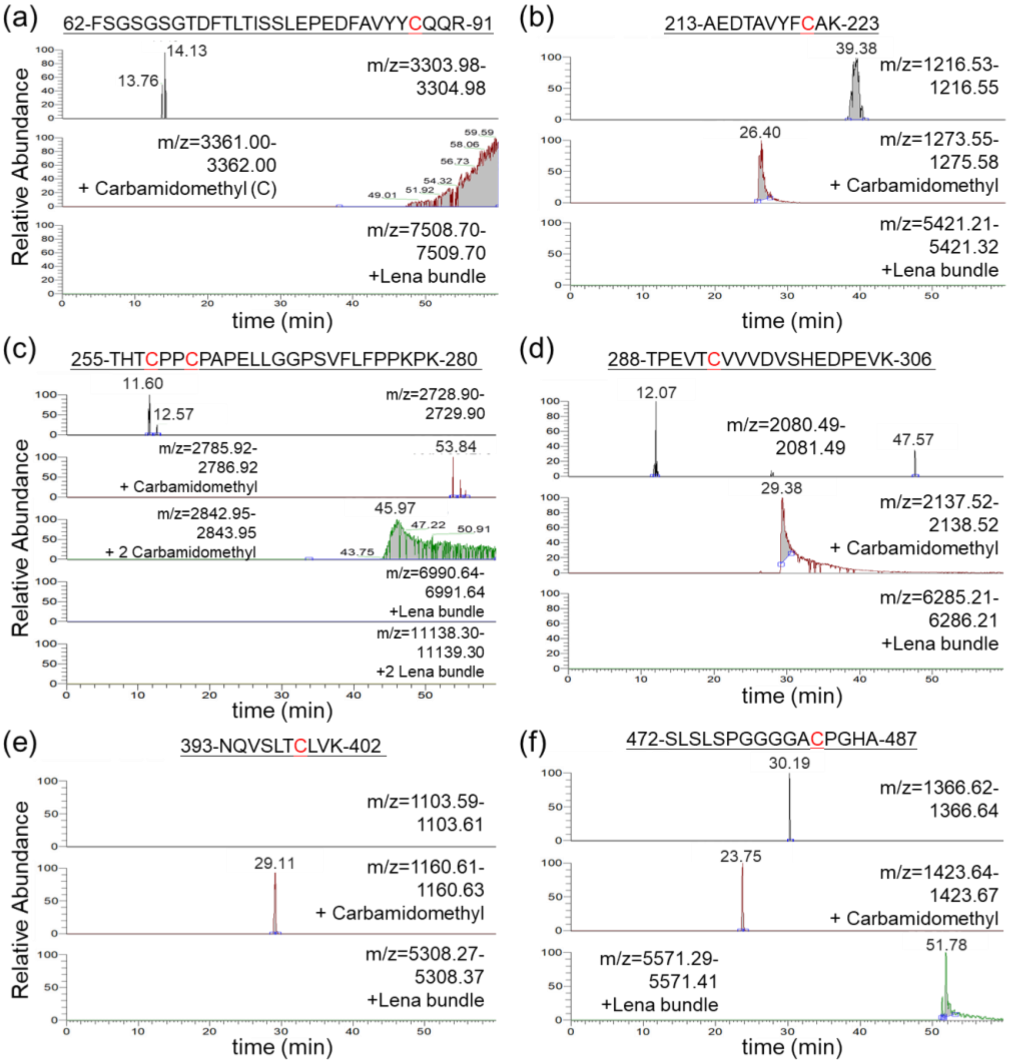

(g)

Peptide Sequence	Theoretical (m/z)	Observed (m/z[a])
^{62}FSGSGSGTDFTLTISSLEPEDFAVYYCQQR^{91}	7,509	ND
^{213}AEDTAVYFCAKD^{223}	5,421	ND
^{255}THTCPPCPAPELLGGPSVFLFPPKPK^{280}	6,991<br>11,138	ND
^{288}TPEVTCVVVDVSHEDPEVK^{306}	6,285	ND
^{393}NQVSLTCLVK^{402}	5,308	ND
^{472}SLSLSPGGGGACPGHA^{487}	5,571	5,571

[a] ND denotes "Not Detectable".

Supplementary Figure S5. Site-specific conjugation of TE-1146 determined by LC-MS. (a-f) The LC-MS results of the trypsin-digested Cys-containing peptides in TE-1146. The numbers at the beginning and end of each peptide sequence indicate the aa number of α-CD38 mAb. In each panel, the MS-scanning region of the predicted molecular weight of each cysteine-containing peptide without the drug bundle is shown at the top; the molecular weight region of the same peptide fragment with an additional carbamoylmethyl adduct (+58 Da) is shown in the middle; whereas that with a lenalidomide drug bundle is shown at the bottom. (g) Theoretical and observed m/z values of Cys-containing peptides+drugs; ND denotes "Not Detectable".

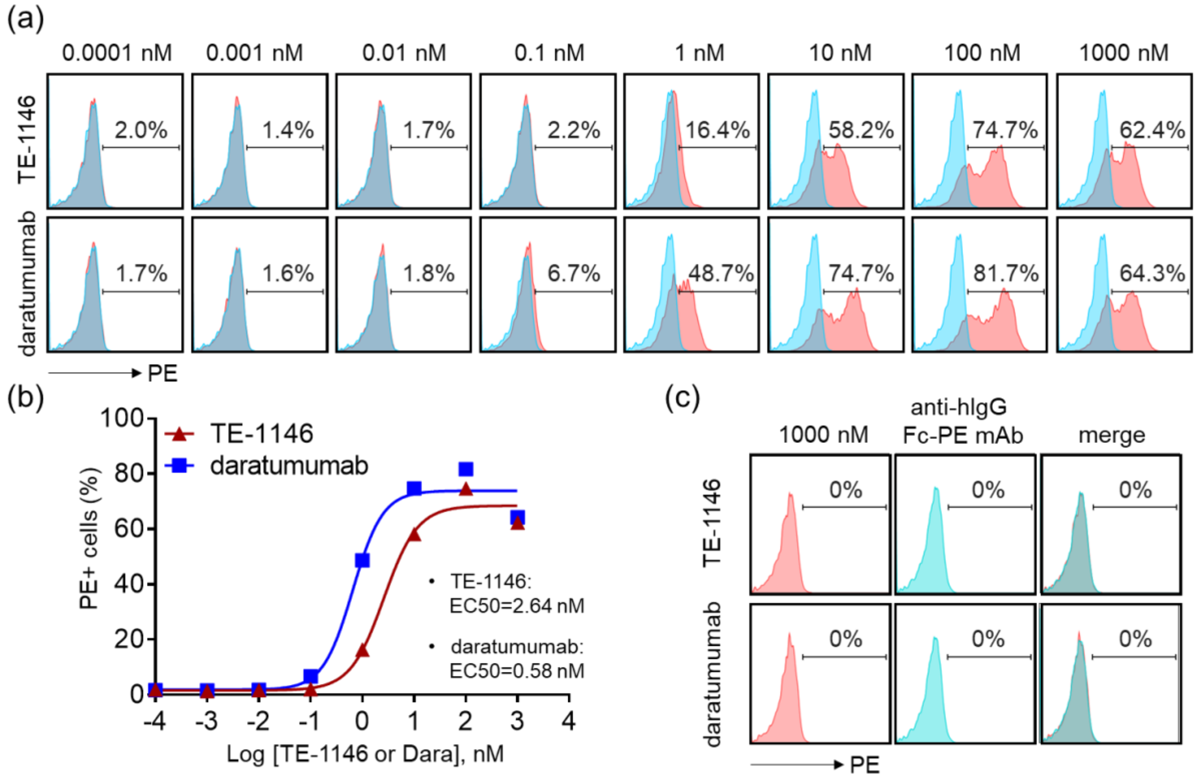

Supplementary Figure S6. *In vitro* cell binding of TE-1146. (a) The binding of TE-1146 and daratumumab to cell surface-expressed CD38 on MM.1S cells was characterized by flow cytometry. (b) A nonlinear regression of flow results from (a) is shown. The EC_{50} values of TE-1146 and daratumumab binding to MM.1S cells are 2.64 nM and 0.58 nM, respectively. (c) The binding of TE-1146 and daratumumab to U266-$CD38^{-}$ MM cell line.

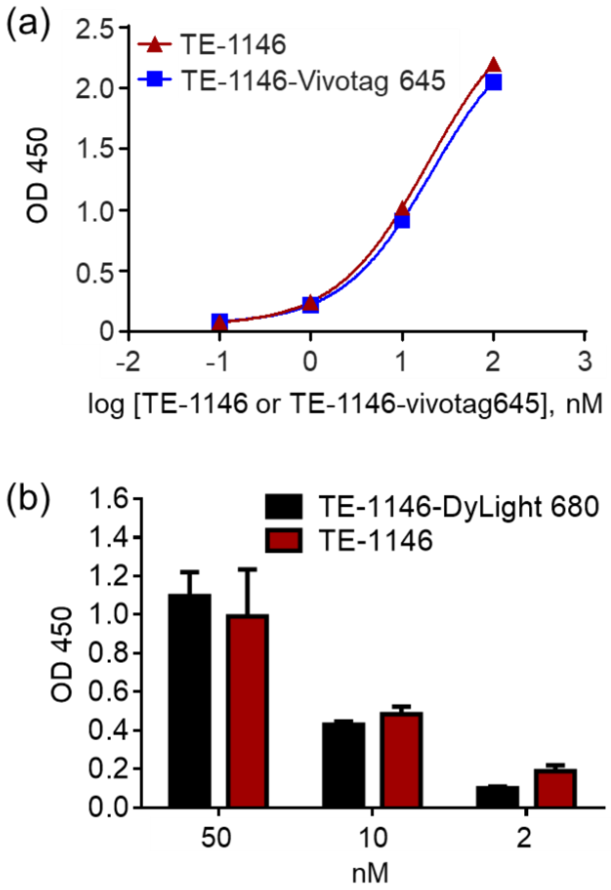

Supplementary Figure S7. Binding activities of TE-1146 and modified TE-1146 using cell-based ELISA. (a) The binding activity of TE-1146 and TE-1146 labeled with VivoTag 645. (b) The binding activities of DyLight^{TM} 680-labeled and unlabeled TE-1146 show no significant difference.

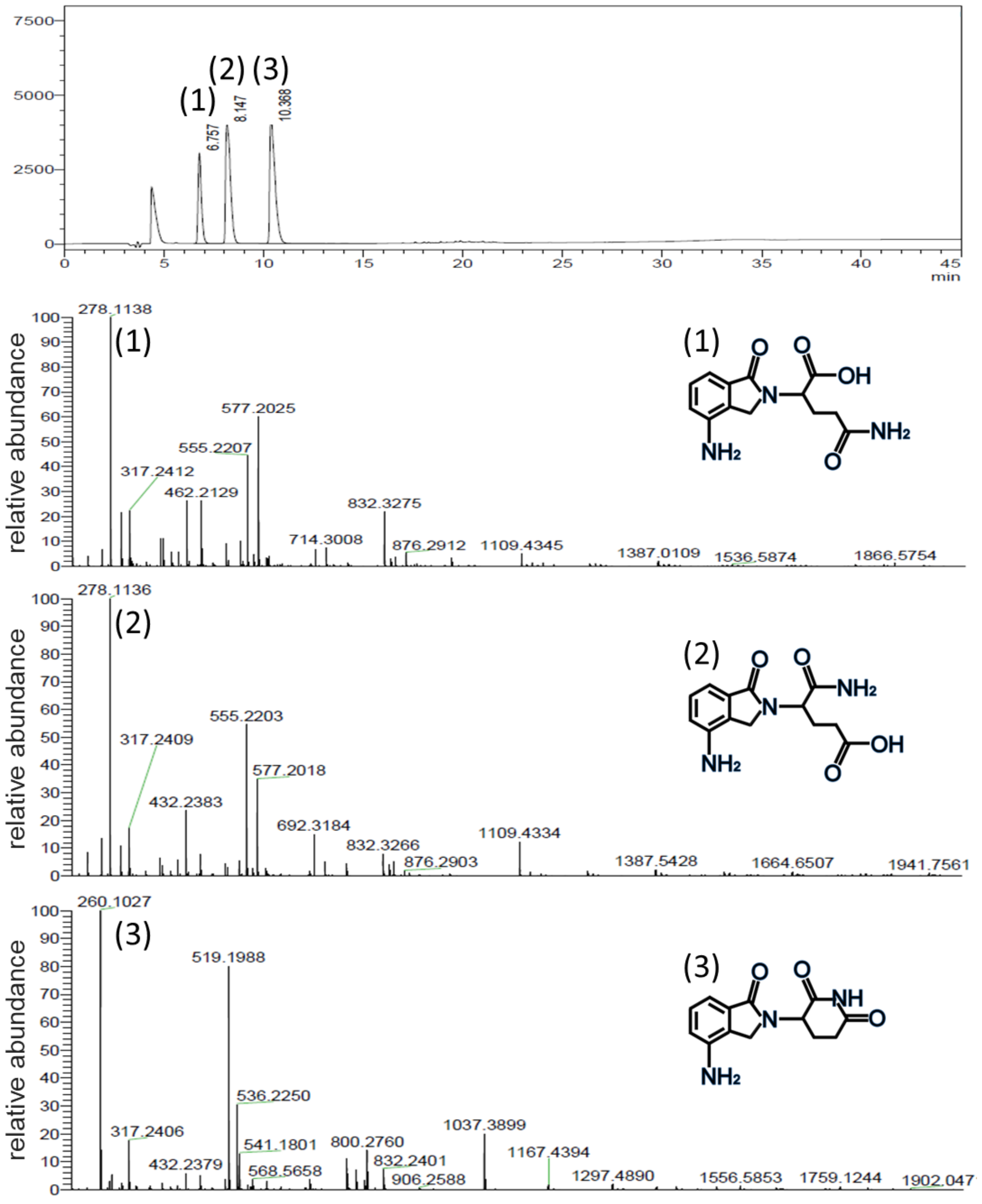

Supplementary Figure S8. Molecular weights of lenalidomide and its two metabolites. LC/MS analysis of the molecular weights of the two metabolites of lenalidomide, marked as (1) and (2), and original lenalidomide with a molecular weight of 260 Da, marked as (3). The structures of the two metabolites were predicted by NMR.

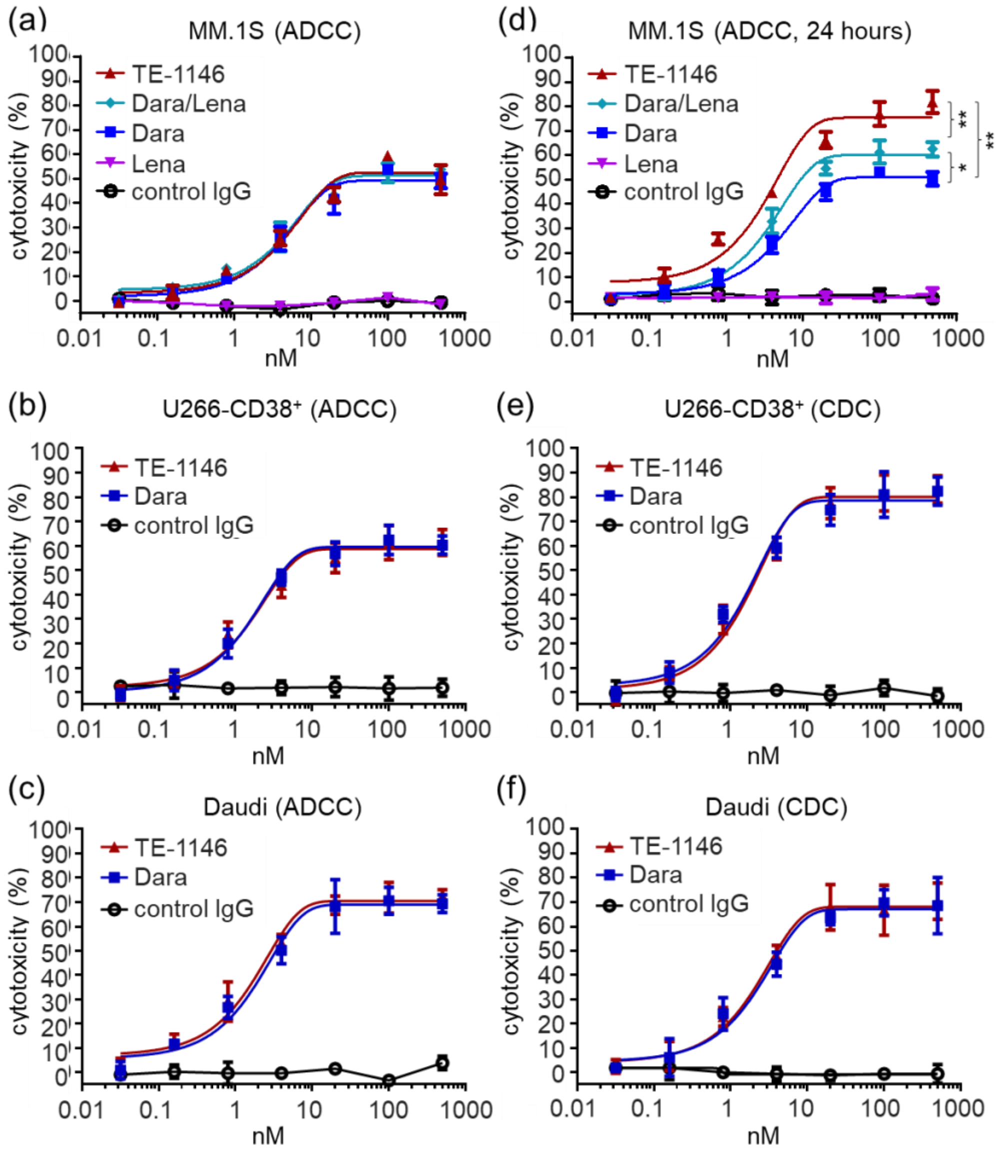

Supplementary Figure S9. TE-1146 induces ADCC and CDC against MM cells *in vitro*. With short-time (5 hours) incubation with MM.1S cells (a), U266-CD38$^+$ cells (b), and Daudi cells (c), TE-1146 and daratumumab alone or combined with lenalidomide show comparable ADCC activities. Control IgG was used as the negative control. (d) With long-term incubation, TE-1146 shows better ADCC effect than daratumumab alone or combined with lenalidomide. With 5 hours incubation with U266-CD38$^+$ cells (e) and Daudi cells (f), TE-1146 and daratumumab show comparable CDC activities. Control IgG was used as the negative control. *p < 0.05; **p < 0.01. Data are shown as mean ± SD. Those with no significant differences are not labeled in the figure.

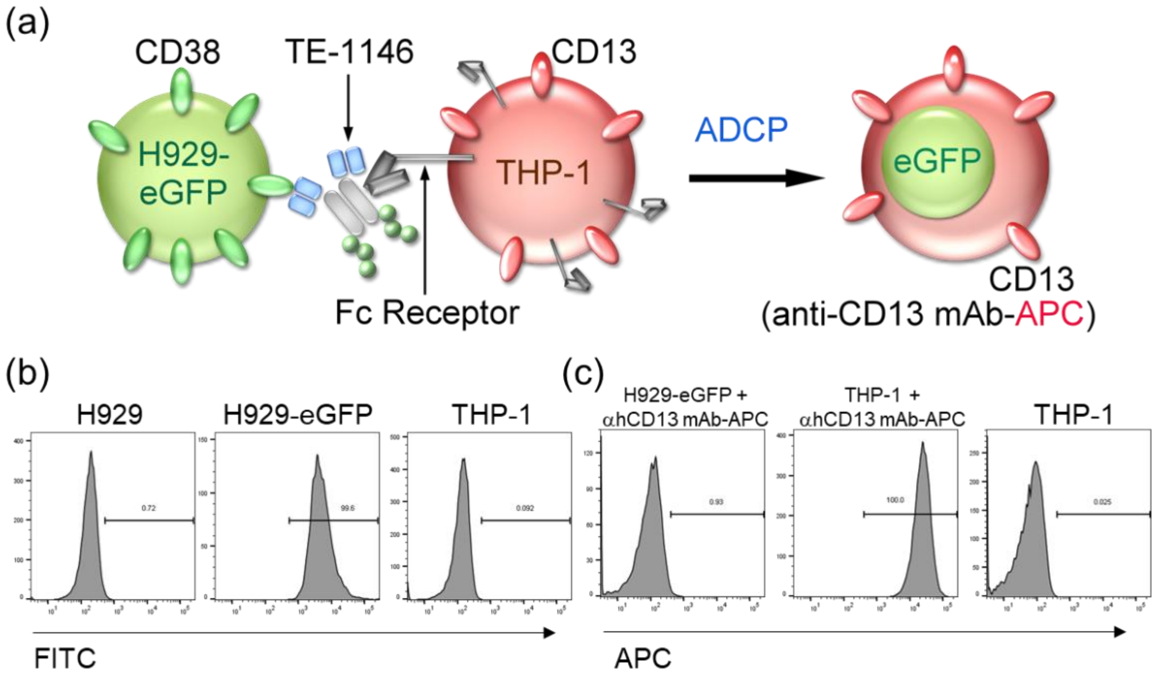

Supplementary Figure S10. TE-1146 induces ADCP against H929-eGFP cells in vitro. (a) The procedure to determine the ADCP activity of TE-1146. The increased amount of eGFP and APC double-positive cells represents relative ADCP activity. (b) eGFP signals of cells used in ADCP assay were determined by flow cytometry. (c) The specificity of the goat anti-human CD13 APC conjugated antibody was determined by flow cytometry.

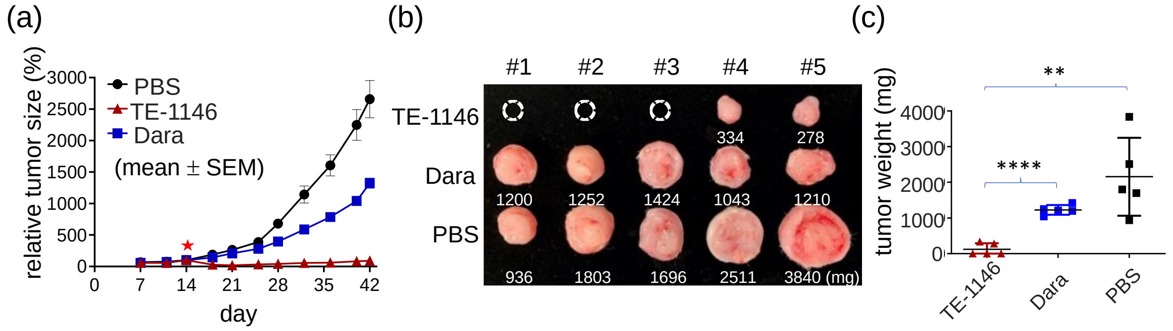

Supplementary Figure S11. *In vivo* efficacy of TE-1146 in MM.1S derived xenograft mouse model. (a) Comparison of TE-1146 monotherapy to daratumumab (Dara) in a MM.1S MM xenograft model (n = 5/group, treated on day 14 post-transplantation marked by the red star). Mice were observed for 28 days post-treatment. (b) Tumors collected from experiments on the final day of observation were weighed and photographed. (c) End-point tumor weight of (b) for the different treatments. Data are shown as mean ± SEM. *p < 0.05; **p < 0.01; ***p < 0.001; ****p < 0.0001. Those with no significant difference are not labeled.

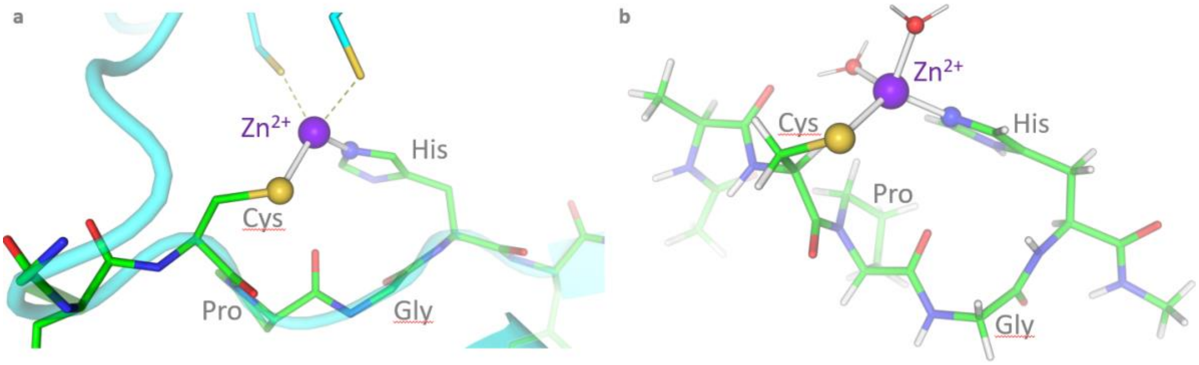

Supplementary Figure S12. Zn^{2+}-binding ACPGH motif. (a) The tetrahedral Zn^{2+}-CCCH site in the 3.30 Å X-ray structure of DNA-directed RNA polymerase subunit from Pyrococcus furiosus (PDB 3qqc) where Zn^{2+} is bound to Cys121, Cys124 as well as Cys131 and His134 of the **CPGH** motif. (b) The Zn^{2+}-binding motif is modeled by replacing the first two Cys in the Zn^{2+}-CCCH site with water molecules to prevent non-specific conjugation of the drug bundle.

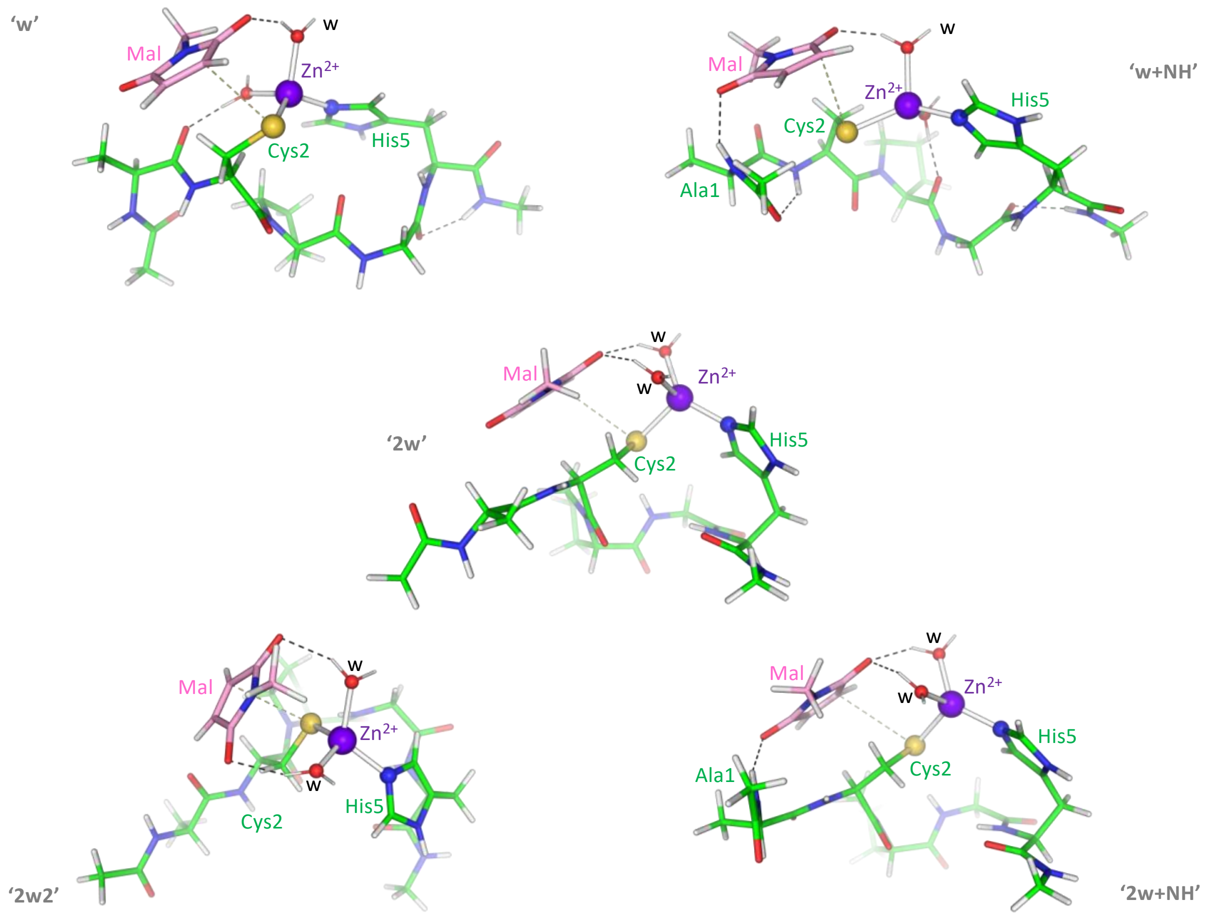

Supplementary Figure S13. Maleimide interacts with Zn^{2+}–ACPGH in different conformations.

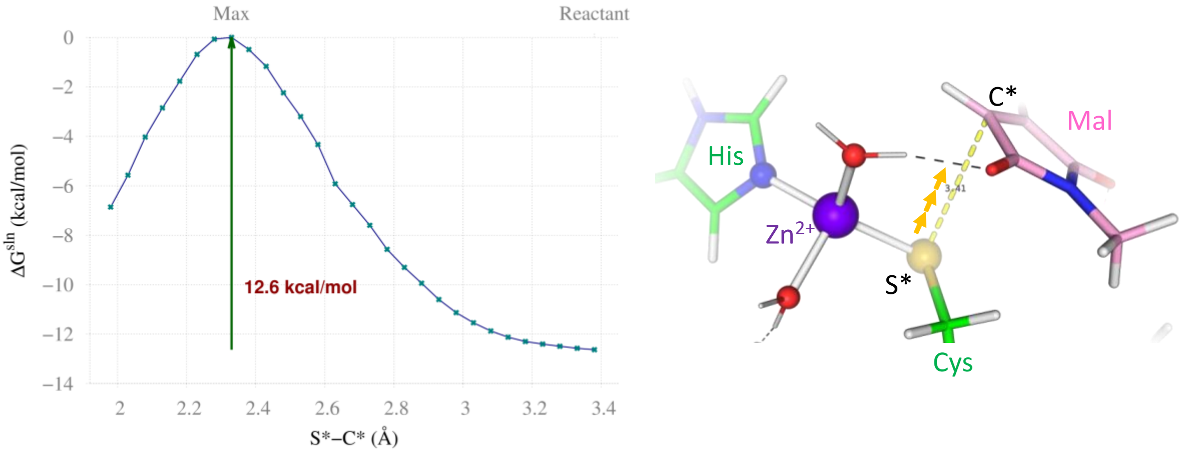

Supplementary Figure S14. **Estimating the reaction barrier.** The solution free energy, ΔG^{sln}, of a given Zn^{2+}–CHww ---Mal conformation as a function of the S*---C* distance, shown on the right.

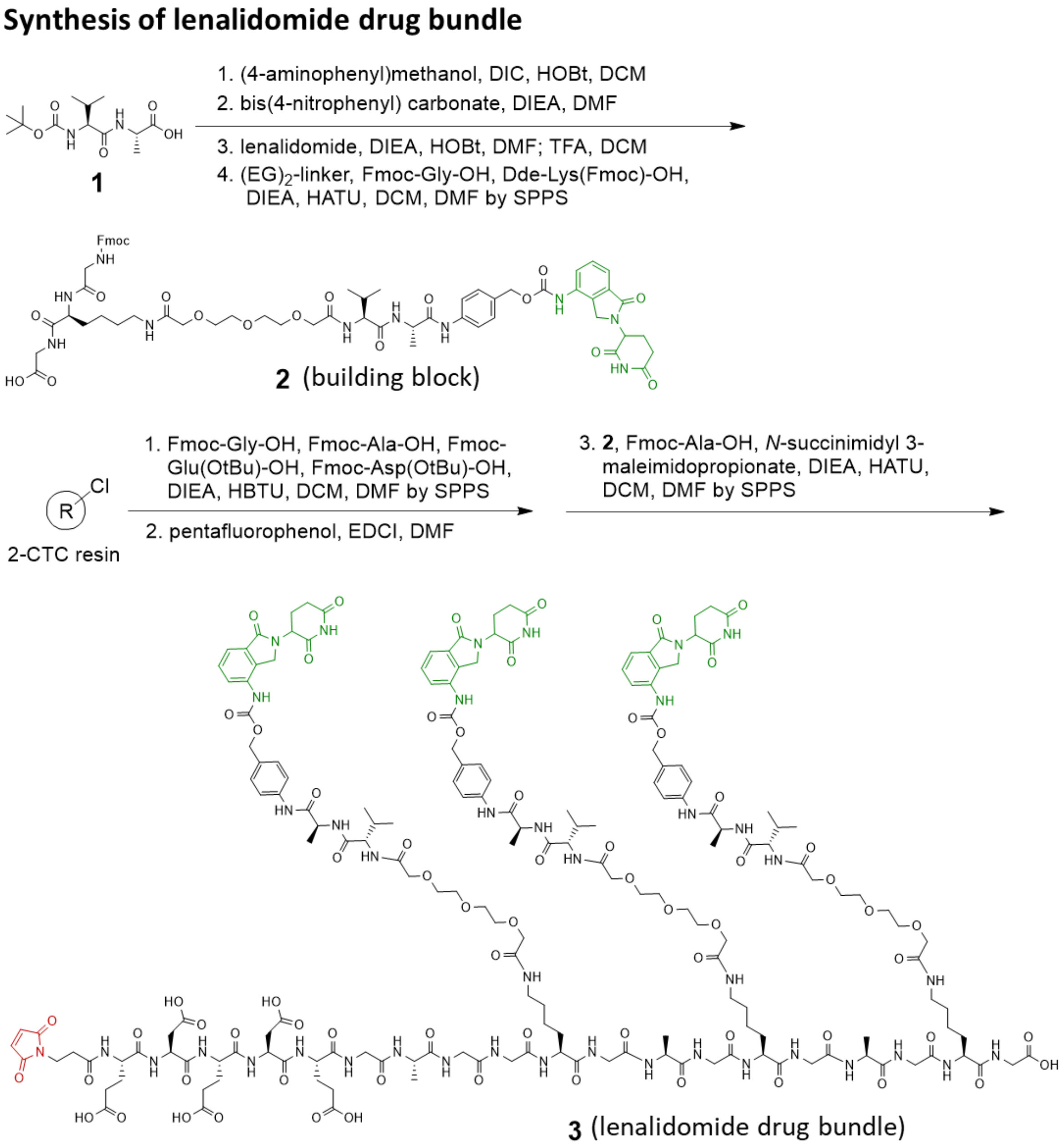

Supplementary Figure S15. Reaction scheme for the synthesis of a lenalidomide drug bundle. First, (4-aminophenyl)methanol, bis(4-nitrophenyl) carbonate, and lenalidomide was sequentially coupled to Boc-protected Val-Ala dipeptide **1** in solution. This was followed by removal of the Boc-protecting group, coupling to a EG linker, then to the Lys ε-amino group of Fmoc-protected Gly-Lys-Gly tripeptide via solid-phase peptide synthesis (SPPS) to yield a building block comprising Gly-Lys-Gly-(EG-linker)-Val-Ala-PABC-lenalidomide (**2**). Next, an EDEDEGAG peptide was synthesized by standard Fmoc-based SPPS and coupled with 2,3,4,5,6-pentafluorophenol at the C-terminus. The resulting activated peptide, along with building block **2**, Fmoc-Ala-OH, and *N*-succinimidyl 3-maleimidopropionate were used to assemble the lenalidomide drug bundle **3** via SPPS. Lenalidomide is in green and maleimide

in red. DCM: Dichloromethane; Dde: 1-(4,4-dimethyl-2,6-dioxocyclohex-1-ylidene)ethyl; DIC: *N*,*N*'-Diisopropylcarbodiimide; DIEA: *N*,*N*-Diisopropylethylamine; DMF: *N*,*N*-Dimethylformamide; $(EG)_2$-linker: 2,2'-((oxybis(ethane-2,1-diyl))bis(oxy))diacetic acid; EDCI: 1-Ethyl-3-(3-dimethylaminopropyl)carbodiimide; Fmoc: 9-Fluorenylmethoxycarbonyl; HATU: Hexafluorophosphate azabenzotriazole tetramethyl uronium; HBTU: Hexafluorophosphate benzotriazole tetramethyl uronium; HOBt: 1-Hydroxybenzotriazole; lenalidomide: 3-(4-amino-1-oxoisoindolin-2-yl)piperidine-2,6-dione; tBu: tert-butyl; TFA: Trifluoroacetic acid

Synthesis of building block 2

First, a solution of (4-aminophenyl)methanol (11.3 g, 91.9 mmol, 1.00 eq), DIC (11.60 g, 91.9 mmol, 14.2 mL, 1.00 eq) and HOBt (12.4 g, 91.9 mmol, 1.00 eq) was added to a solution of Boc-protected Val-Ala dipeptide **1** (26.5 g, 91.9 mmol, N/A purity, 1.00 eq) in DCM (300 mL). The mixture was stirred at 20°C for 2 hours. The crude peptide was concentrated under reduced pressure and then purified by flash chromatography using a gradient of water and acetonitrile (A: H_2O, B: acetonitrile). This yielded Boc-Val-Ala-para-aminobenzyl (PAB)-OH (22.4 g, crude) as a white solid. The theoretical and observed m/z values of the $[M+H]^+$ ion were 394.48 and 394.4, respectively.

Next, bis(4-nitrophenyl) carbonate (18.9 g, 62.2 mmol, 2.00 eq) and DIEA (16.1 g, 124 mmol, 21.7 mL, 4.00 eq) were added to a solution of Boc-Val-Ala-PAB-OH (12.25 g, 31.1 mmol, N/A purity, 1.00 eq) in DMF (100 mL). The mixture was stirred at 20°C for 2 hours. The crude peptide was concentrated under reduced pressure and purified by flash chromatography (A: H_2O, B: acetonitrile), yielding Boc-Val-Ala-PAB-p-nitrophenol carbonate (16.3 g, crude) as a white solid.

Lenalidomide (11.3 g, 43.7 mmol, 1.50 eq), DIEA (3.77 g, 29.2 mmol, 5.08 mL, 1.00 eq) and HOBt (1.97 g, 14.6 mmol, 0.50 eq) were added to a solution of Boc-Val-Ala-PAB-p-nitrophenol carbonate (16.3 g, 29.1 mmol, N/A purity, 1.00 eq) in DMF (200 mL). The mixture was stirred at 40°C for 72 hours. The crude peptide was then purified by prep-HPLC (A: 0.075% TFA in H_2O, B: acetonitrile), yielding Boc-Val-Ala-para-aminobenzylcarbamate (PABC)-lenalidomide (4.53 g, 3.90 mmol, 13.3% yield, 68.22% purity, TFA) as a white solid; the theoretical and observed m/z values of the $[M+H]^+$ ion were 679.74 and 679.5, respectively. To remove the BOC protecting group, a solution of Boc-Val-Ala-PABC-lenalidomide (4.53 g, 3.90 mmol, 68.2% purity, 1.00 eq TFA) in TFA (20.0 mL) and DCM (20.0 mL) was stirred at 25°C for 30 minutes. The crude peptide was then concentrated under reduced pressure and precipitated with cold isopropyl ether (100 mL), yielding Val-Ala-PABC-lenalidomide (3.00 g, crude, TFA) as a white solid; the theoretical and observed m/z values of the $[M+H]^+$ ion are 579.63 and 579.4, respectively.

2,2'-((oxybis(ethane-2,1-diyl))bis(oxy))diacetic acid (100 mmol, 22.2 g, 10.0 eq) and DIEA (40.0 mmol, 6.97 mL, 4.00 eq) in DCM (60.0 mL) were added to the 2-CTC resin (10.0 mmol, 1.00 eq, Sub 1.10 mmol/g). The mixture was agitated with N_2 at 20°C for 12 hours, then MeOH (10.0 mL) was added and agitated again with N_2 for another 30 minutes. The resin was washed and filtrated with DMF. A solution of Val-Ala-PABC-lenalidomide (1.60 g, crude, TFA), DIEA (0.94 mL) and HATU (1.02 g) in DMF (60.0 mL) was added to the resin and agitated with N_2 at 20°C for 30 minutes. The resin was washed with DMF followed by DCM and dried under vacuum. Then 80.0 mL of cleavage buffer (20% hexafluoroisopropanol, 80%

DCM) was added to the flask containing the side chain-protected peptide resin at 20°C and stirred for 20 minutes twice. The crude peptide was purified by prep-HPLC (A: 0.075% TFA in H_2O, B: acetonitrile), yielding HOOC-CH_2O-(CH_2CH_2O)$_2$-CH_2CO-Val-Ala-PABC-lenalidomide (0.58 g, 576 μmol, 5.76% yield, 77.7% purity). The theoretical and observed m/z values of the $[M+H]^+$ ion are 783.8 and 783.5, respectively.

A solution of Fmoc-Gly-OH (3.00 mmol, 892 mg, 1.00 eq) and DIEA (12.0 mmol, 2.01 mL, 4.00 eq) in DCM (20.0 mL) was added to the 2-CTC resin (3.00 mmol, 1.00 eq, Sub 1.10 mmol/g). The mixture was agitated with N_2 at 20°C for 2 hours, then MeOH (3.00 mL) was added and agitated again with N_2 for another 30 minutes. The resin was washed and filtrated with DMF. To remove the Fmoc-protecting group, 20% piperidine in DMF (40.0 mL) was added. The resin was agitated with N_2 for another 30 minutes, and washed with DMF and filtered. A solution of Dde-Lys(Fmoc)-OH (6.00 mmol, 2.40 g, 1.50 eq), DIEA (9.00 mmol, 1.57 mL, 3.00 eq) and HATU (4.26 mmol, 1.62 g, 1.42 eq) in DMF (20.0 mL) was added to the resin and agitated with N_2 at 20°C for 30 minutes. The resin was then washed and filtrated with DMF. 20% piperidine in DMF (40.0 mL) was added and the resin was agitated with N_2 for another 30 minutes. A solution of HOOC-CH_2O-(CH_2CH_2O)$_2$-CH_2CO-Val-Ala-PABC-lenalidomide (0.58 g), DIEA (0.26 mL) and HATU (281 mg) in DMF (20.0 mL) was added to the resin and agitated with N_2 at 20°C for 60 minutes. The resin was then washed and filtrated with DMF. 3% hydrazine hydrate/DMF was added and reacted for 10 minutes. It was then drained and the resin washed with DMF. A solution of Fmoc-Gly-OH (9.00 mmol, 2.67 g, 3.00 eq), DIEA (18.0 mmol, 3.13 mL, 6.00 eq) and HATU (8.55 mmol, 3.24 g, 2.85 eq) in DMF (20.0 mL) was added to the resin and agitated with N_2 at 20°C for 30 v. The resin was washed with DCM and dried under vacuum. Then 40.0 mL of cleavage buffer (20% hexafluoroisopropanol, 80% DCM) was added to the flask containing the side chain protected peptide resin at 20°C and stirred for 20 minutes twice. The peptide was concentrated under reduced pressure and purified by prep-HPLC (A: 0.075% TFA in H_2O, B: acetonitrile) to give building block **2** (362.6 mg, 279 μmol, 9.33% yield, 96.29% purity), obtained as a white solid. The theoretical and observed m/z values of the $[M+H]^+$ ion are 1248.33 and 1247.8 respectively. The resulting building block **2** was structurally characterized by ^1H NMR (Bruker AVANCE III HD 400 MHz) and ^{13}C NMR (Bruker AVANCE NEO 400 MHz) (Figures S16 and S17).

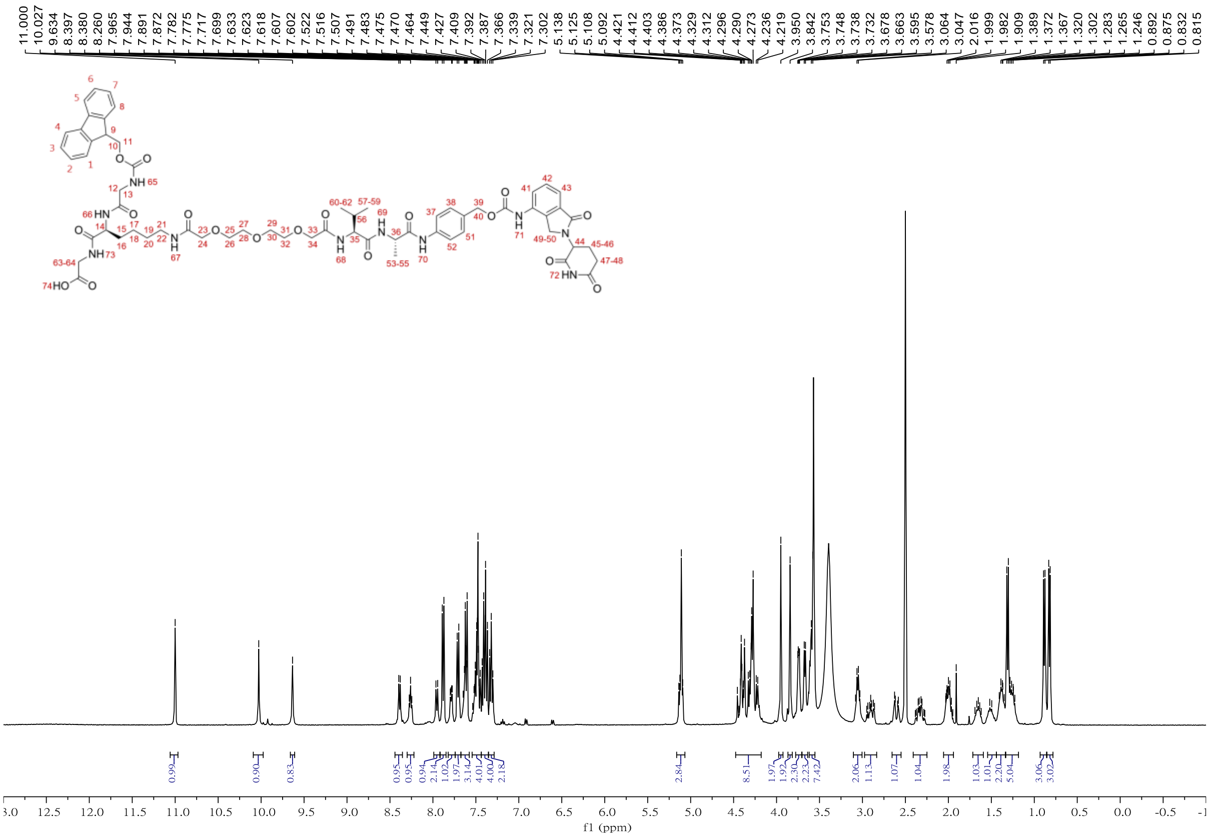

Supplementary Figure S16. ^{1}H spectrum of building block 2 (400 MHz, 298K, DMSO-d6) and peak assignments. δ 11.00 (s, 1H, H-72), 10.03 (s, 1H, H-71), 9.63 (s, 1H, H-70), 8.39 (d, *J* = 6.9 Hz, 1H, H-73), 8.26 (t, *J* = 5.8 Hz, 1H, H-69), 7.95 (d, *J* = 8.2 Hz, 1H, H-68), 7.88 (d, *J* = 7.5 Hz, 2H, H-4/H-5), 7.79 (dd, *J* = 6.1, 2.5 Hz, 1H, H-66), 7.71 (d, *J* = 7.4 Hz, 2H, H-37/H-52), 7.61 (d, *J* = 8.5 Hz, 3H, H-41/H-43/H-67), 7.55 – 7.44 (m, 4H, H-3/H-6/H-42/H-65), 7.40 (q, *J* = 8.9, 8.1 Hz, 4H, H-1/H-8/H-51/H-38), 7.32 (t, *J* = 7.4 Hz, 2H, H-2/H-7), 5.12 (q, *J* = 6.5, 5.8 Hz, 3H, H-10/H-11/H-44), 4.48 – 4.19 (m, 9H, H-9/H-35/H-36/H-49/H-50/H-63/H-64), 3.95 (s, 2H, H-39/H-40), 3.84 (s, 2H, H-23/H-24), 3.74 (d, *J* = 5.9 Hz, 2H, H-29/H-31), 3.67 (d, *J* = 6.0 Hz, 2H, H-25/H-27), 3.63 – 3.53 (m, 8H, H-12/H-13/H-26/H-28/H-30/H-32/H-33/H-34), 3.09 – 3.01 (m, 2H, H-21/H-22), 2.90 (td, *J* = 13.5, 6.8 Hz, 1H, H-46), 2.60 (d, *J* = 17.2 Hz, 1H, H-45), 2.33 (dd, *J* = 13.0, 4.4 Hz, 1H, H-56), 2.04 – 1.94 (m, 2H, H-47/H-48), 1.66 (d, *J* = 7.0 Hz, 1H, H-16), 1.55 – 1.47 (m, 1H, H-15), 1.41 – 1.35 (m, 2H, H-19/H-20), 1.34 – 1.21 (m, 5H, H-17/H-18/H-53/H-54/H-55), 0.88 (d, *J* = 6.7 Hz, 3H, H-60/H-61/H-62), 0.82 (d, *J* = 6.7 Hz, 3H, H-57/H-58/H-59) ppm.

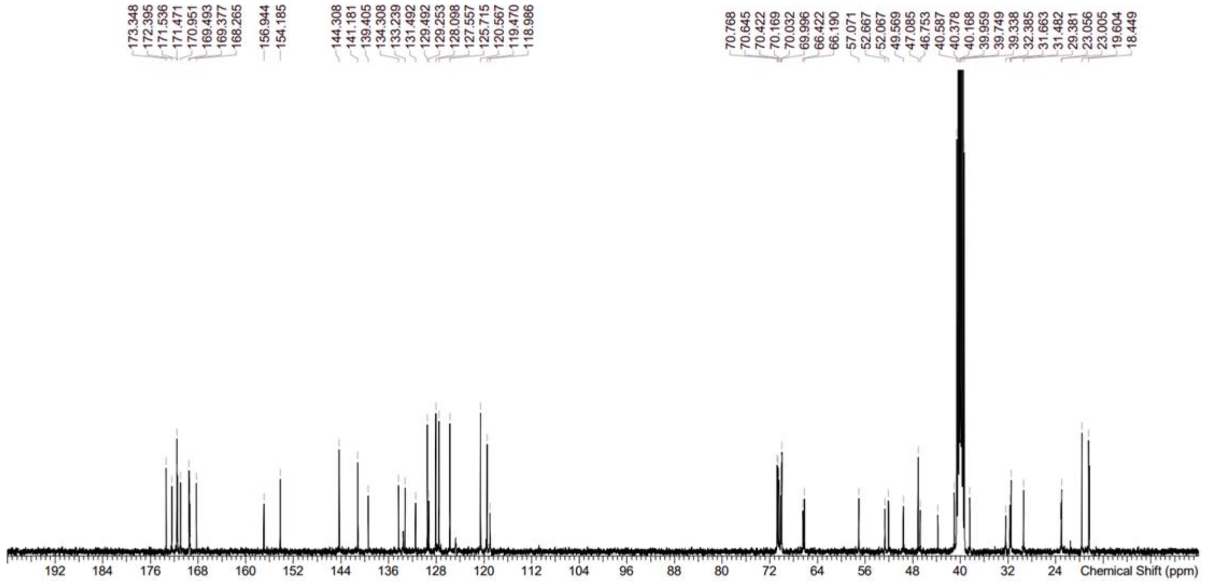

Supplementary Figure S17. ^{13}C-NMR spectrum of building block 2 (101 MHz, 298K, DMSO-d6) and peak assignments. δ = 173.35, 172.4, 171.57, 171.54, 171.48, 170.95, 169.5, 169.45, 169.38, 168.27, 156.95, 154.19, 144.31, 141.18, 139.41, 134.31, 133.54, 133.24, 131.5, 129.5, 129.5, 129.26, 128.1, 128.1, 127.56, 127.56, 125.72, 125.72, 124.76, 120.57, 120.57, 119.47, 119.47, 118.99, 70.77, 70.65, 70.43, 70.17, 70.04, 70, 66.42, 66.19, 57.07, 52.67, 52.07, 49.57, 47.09, 46.76, 43.78, 41.03, 38.42, 32.39, 31.67, 31.49, 29.38, 23.06, 23.01, 19.61, 18.45, 18.36 ppm.

Assembly of lenalidomide drug bundle using building block 2 and EDEDEGAG peptide

The EDEDEGAG peptide was synthesized using standard Fmoc chemistry. Fmoc-Gly-OH (5.00 mmol, 1.49 g, 1.00 eq) and DIEA (20.0 mmol, 3.48 mL, 4.00 eq) in DCM (30.0 mL) were added to the 2-CTC resin (5.00 mmol, 1.00 eq, Sub 1.10 mmol/g). The mixture was agitated with N_2 at 20°C for 2 hours, then MeOH (5.00 mL) was added and agitated with N_2 for another 30 minutes. The resin was washed and filtrated with DMF. to remove the protecting group, 20% piperidine in DMF (60.0 mL) was added. The resin was agitated with N_2 for another 30 minutes, then washed with DMF and filtered. A solution of Fmoc-Ala-OH (15.0 mmol, 4.67 g, 3.00 eq), DIEA (30.0 mmol, 5.23 mL, 6.00 eq) and HBTU (14.2 mmol, 5.41 g, 2.85 eq) in DMF (30.0 mL) was added to the resin and agitated with N_2 at 20°C for 30 minutes. The resin was then washed and filtrated with DMF. The deprotection and coupling steps were repeated for the subsequent coupling of Fmoc-Gly-OH, Fmoc-Glu(OtBu)-OH and Fmoc-Asp(OtBu)-OH. The resin was washed with MeOH and dried under vacuum. Then 80.0 mL of cleavage buffer (1% TFA, 99% DCM) was added to the flask containing the side chain-protected peptide resin at room temperature and stirred for 5 minutes twice. The crude peptide was concentrated under reduced pressure to give tBu-protected Fmoc-[EDEDE]-GAG (6.23 g, crude), obtained as a white solid. The theoretical and observed m/z values of the $[M+H]^+$ ion are 1324.5 and 1323.9.

2,3,4,5,6-pentafluorophenol, abbreviated as PFP (834 mg, 4.53 mmol, 1.50 eq) and EDCI (869 mg, 4.53 mmol, 1.50 eq) were added to a solution of tBu-protected Fmoc-[EDEDE]-GAG (4.00 g, 3.02 mmol, N/A purity, 1.00 eq) in DMF (30.0 mL). The mixture was stirred at 20°C for 2 h and concentrated under reduced pressure. Then 30 mL of cleavage buffer (92.5% TFA, 2.5% triisopropylsilane, 2.5% H_2O, 2.5% 3-mercaptopropionic acid) was added to the flask containing the side chain-protected peptide at 20°C and stirred for 1 hour. The crude peptide was precipitated with cold isopropyl ether (200 mL) and then filtered to collect the filter cake. The crude peptide was purified by prep-HPLC (A: 0.075% TFA in H_2O, B: acetonitrile), yielding Fmoc-EDEDEGAG-PFP (1.13 g, 291 μmol, 9.63% yield, 31.15% purity) as a white solid. The theoretical and observed m/z values of the $[M+H]^+$ ion are 1210.01 and 1209.6.

The lenalidomide drug bundle was assembled by coupling building block 2 with the tBu-protected Fmoc-EDEDEGAG-PFP peptide. A solution of building block **2** (100 mg) and DIEA (1.20 mmol, 0.21 mL, 4.00 eq) in DCM (1.50 mL) and DMF (0.20 mL) was added to the 2-CTC resin (0.30 mmol, 1.00 eq, Sub 1.10 mmol/g). The mixture was agitated with N_2 at 20°C for 3 hours, then MeOH (0.30 mL) was added and agitated with N_2 for another 30 minutes. The resin was washed and filtrated with DMF. 20% piperidine in DMF (4.00 mL) was added. The resin was agitated with N_2 for another 30 minutes, then washed and filtrated with DMF. A solution of Fmoc-Ala-OH (0.90 mmol, 280 mg, 3.00 eq), DIEA (1.80 mmol, 0.31 mL, 6.00 eq) and HATU (0.85 mmol, 324 mg, 2.85 eq) in DMF (2.00 mL) was added to the resin and agitated with N_2 at 20°C for 30 minutes. The resin was then washed and filtrated with DMF. The

deprotection and coupling steps were repeated for the subsequent coupling of building block **2** and tBu-protected Fmoc-EDEDEGAG-PFP, Fmoc-Ala-OH, and *N*-succinimidyl 3-maleimidopropionate. The resin was washed with DCM and dried under vacuum. Then 6.00 mL of cleavage buffer (20% hexafluoroisopropanol, 80% DCM) was added to the flask containing the side chain-protected peptide resin at 20°C and stirred for 20 minutes twice. The crude peptide was concentrated under reduced pressure and then purified by prep-HPLC (A: 0.075% TFA in H_2O, B: acetonitrile), yielding the final lenalidomide drug bundle (16.1 mg, 3.77 μmol, 1.26% yield, 96.71% purity) as a white solid. The observed m/z of the $[M+3H]^{3+}$ ion (1379.24) matched the theoretical value, 1379.4.
